# Management of Acute Myeloid Leukemia: Current Treatment Options and Future Perspectives

**DOI:** 10.3390/cancers13225722

**Published:** 2021-11-16

**Authors:** Maximilian Fleischmann, Ulf Schnetzke, Andreas Hochhaus, Sebastian Scholl

**Affiliations:** Klinik für Innere Medizin II, Abteilung Hämatologie und Onkologie, Universitätsklinikum Jena, Am Klinikum 1, 07740 Jena, Germany; Maximilian.Fleischmann@med.uni-jena.de (M.F.); Ulf.Schnetzke@med.uni-jena.de (U.S.); Andreas.Hochhaus@med.uni-jena.de (A.H.)

**Keywords:** AML, targeted therapy, clinical trial, resistance

## Abstract

**Simple Summary:**

AML is a genetically heterogeneous disease with a median age of diagnosis between 60 and 70 years. Thus, many AML patients are not eligible for intensive chemotherapy. Often, the disease is accompanied by a poor prognosis due to high-risk genetic features or due to antecedent hematologic disorders (e.g., myelodysplastic syndrome). Therefore, AML treatment remains a challenge; even after intensive chemotherapy and allogeneic stem cell transplantation (alloHSCT), AML relapses are regularly observed. Thus, new concepts of AML therapy, considering tailored treatment approaches after comprehensive molecular diagnostic or implementing new immunotherapeutic strategies, are urgently needed. This review provides a detailed overview of recent developments and current promising concepts to improve the treatment and the outcome of AML patients.

**Abstract:**

Treatment of acute myeloid leukemia (AML) has improved in recent years and several new therapeutic options have been approved. Most of them include mutation-specific approaches (e.g., gilteritinib for AML patients with activating *FLT3* mutations), or are restricted to such defined AML subgroups, such as AML-MRC (AML with myeloid-related changes) or therapy-related AML (CPX-351). With this review, we aim to present a comprehensive overview of current AML therapy according to the evolved spectrum of recently approved treatment strategies. We address several aspects of combined epigenetic therapy with the BCL-2 inhibitor venetoclax and provide insight into mechanisms of resistance towards venetoclax-based regimens, and how primary or secondary resistance might be circumvented. Furthermore, a detailed overview on the current status of AML immunotherapy, describing promising concepts, is provided. This review focuses on clinically important aspects of current and future concepts of AML treatment, but will also present the molecular background of distinct targeted therapies, to understand the development and challenges of clinical trials ongoing in AML patients.

## 1. Introduction

Acute myeloid leukemia (AML) is a heterogenous disease with a broad spectrum of cytogenetic and molecular aberrations contributing to the definition of distinct AML subgroups. Treatment options for patients suffering from AML are continuously expanding and targeted therapies are available for distinct molecularly defined subgroups. Nevertheless, AML treatment remains challenging; in particular, patients with high-risk AML not eligible for intensive treatment or allogeneic hematopoietic stem cell transplantation (alloHSCT) are characterized by an unfavorable outcome.

The occurrence of AML relapse is attributed to the persistence and clonal evolution of leukemic stem cells (LSCs). To date, the approval of AML-targeted therapy is mostly restricted to elderly AML patients or relapsed or refractory AML (r/r AML), while only a minority of patients who are refractory to chemotherapy subsequently undergo potential curative alloHSCT. Thus, current strategies of AML precision medicine aim to target the LSC compartment, to allow longer remission and to provide the chance of further consolidation treatment. 

This review provides a comprehensive survey of both current concepts of AML therapy and promising approaches currently being investigated in clinical trials or awaiting approval by the Food and Drug administration (FDA) or European Medicines Agency (EMA). We discuss neither present treatment strategies of acute promyelocytic leukemia (APL) nor recent advances of conventional chemotherapy, including liposomal formulation of CPX-351 (Vyxeos).

Furthermore, we address potential mechanisms of resistance observed in venetoclax-based AML regimens and provide insights into the recent progress to overcome resistance by addressing different cellular targets. We further describe current development of immunotherapy in AML in detail and present an overview of targeted therapies and immunotherapeutic approaches that are characterized by their potential to effectively address LSCs of AML.

## 2. FLT3 Mutations in AML

### 2.1. General Aspects of FLT3 Mutations and Treatment

Activating mutations of the FMS-like tyrosine kinase 3 (*FLT3*) occurring in about 30% of newly diagnosed AML play a pivotal role in diagnostic algorithms, prognostic stratification, and first line treatment of AML patients. Besides the detection of the most frequent and almost patient-specific *FLT3-ITD* (FLT3-internal tandem duplication) that can be found in approximately 25% of all AML patients, activating mutations located in the *FLT3-TKD* (FLT3-tyrosine kinase domain) are found in about 7% of patients at diagnosis [1,2]. Both the European LeukemiaNet (ELN) and the National Comprehensive Cancer Network (NCCN) do not consider the presence of *FLT3-TKD* mutations as a recommendation for alloHSCT since they are not associated with a poor prognosis in general [3,4]. Thus, all patients with *FLT3-TKD* mutations completing intensive induction and consolidation chemotherapy should receive maintenance treatment with midostaurin for 48 weeks [5,6].

In context of a normal karyotype, prognostic stratification of *FLT3-ITD* is more complex. It depends on the co-occurrence of nucleophosmin 1 (*NPM1*) mutations and is affected by the mutant-to-wild-type allelic ratio (AR) of *FLT3-ITD,* according to the ELN 2017 guidelines. The discrimination between *FLT3-ITD* “low” vs. “high” in terms of the allelic ratio is defined by a cutoff of 0.5 (e.g., “low” AR < 0.5 and “high” ≥ 0.5). In contrast to the NCCN recommendations, ELN 2017 classification stratifies AML patients with *NPM1mut/FLT3-ITD^low^* as favorable while only those patients with *NPM1wt/FLT3-ITD^high^* are attributed to the adverse prognostic subgroup [3]. 

This prognostic stratification is still under debate and especially patients with *FLT3-ITD^low^* lacking a concomitant *NPM1* mutation are difficult to monitor for minimal residual disease (MRD) and several studies demonstrate a clinical benefit of alloHSCT in first remission of AML patients harboring *FLT3-ITD* independently of the AR of FLT3-ITD [7,8]. 

In general, all patients with activating *FLT3* mutations undergoing intensive chemotherapy (e.g., “7 + 3” induction followed by high-dose cytarabine consolidation) should receive midostaurin for 14 days after each chemotherapy course followed by midostaurin maintenance unless subsequent alloHSCT is indicated [5].

### 2.2. Therapeutic Implications of Distinct FLT3 Mutations

Besides the impact of *FLT3-ITD* AR in presence of a *NPM1* mutation on ELN classification and allocation to conventional consolidation or upfront alloHSCT, the occurrence of *FLT3-TKD* mutation does not implicate alloHSCT in first CR of AML. As described above, patients harboring *FLT3-TKD* mutations are recommended to undergo maintenance therapy with midostaurin for 12 cycles of 4 weeks each. 

Due to the molecular individuality of *FLT3-ITD* comprising almost unique tandem duplications of the *FLT3* gene, many efforts were made to subclassify *FLT3-ITDs* and to understand potential differences in terms of biology and possibly prognosis of distinct *FLT3-ITD* subtypes. Approximately 30% of *FLT3-ITD* are localized within the tyrosine kinase domain 1 (*TKD1*) of *FLT3* being associated with an inferior outcome following intensive AML treatment [9,10]. Breitenbuecher and co-workers were able to demonstrate a potential resistance mechanism of *FLT3-ITD* located in the *TKD1* region towards midostaurin that was caused by aberrant upregulation of the anti-apoptotic protein *MCL-1* (myeloid cell leukemia-1) [11]. 

Recent data obtained from the RATIFY study revealed a significantly different impact of *FLT3-ITD* subtype on the treatment effect of midostaurin during intensive chemotherapy of AML patients. The prognostically relevant heterogeneity of *FLT3-ITD* and the poor outcome of *FLT3-ITD* localization in the *TKD1* region have been confirmed by Rücker and co-workers [12]. This comprehensive retrospective analysis revealed that the beneficial effect of midostaurin is restricted to “classical *FLT3-ITDs*” located in the juxtamembrane domain (*JMD*). Furthermore, the authors demonstrate an improved outcome for patients undergoing alloHSCT following induction chemotherapy in combination with midostaurin.

Considering these results, we postulate several clinical consequences: (1) the *FLT3-ITD* subgroup analysis should be implemented in diagnostic algorithms at diagnosis; (2) midostaurin maintenance therapy needs to be evaluated critically in case of *FLT3-ITD* located within the *TKD1* domain; and (3) when lacking reliable MRD markers in AML patients with a prognostically relevant *FLT3-ITD* subtype, alloHSCT should be considered in first CR.

### 2.3. Maintenance Treatment in FLT3 Mutated AML beyond Midostaurin

Maintenance therapy with midostaurin has been approved by EMA and reflects the standard of care in AML patients harboring either *FLT3-ITD* or *FLT3-TKD* mutations, undergoing conventional induction and consolidation chemotherapy without alloHSCT. All patients randomized to midostaurin treatment at diagnosis within the RATIFY trial were recommended to receive maintenance treatment with midostaurin unless they underwent alloHSCT. Thus, there was no second randomization investigating the impact of midostaurin maintenance alone [5]. 

Several clinical trials addressed the question of *FLT3* inhibitor maintenance therapy after alloHSCT. The placebo-controlled SORMAIN trial could demonstrate a significantly improved survival of AML patients who received sorafenib following alloHSCT [13]. Similar results were obtained from the phase 3 trial randomizing 202 AML patients for either sorafenib or placebo after alloHSCT [14]. Important translational studies revealed a special impact of IL-15, mediating enhanced graft-versus-leukemia effects (GvL) in sorafenib-treated patients after alloHSCT [15]. While sorafenib has not been approved for post-alloHSCT maintenance, gilteritinib can be reapplied in this clinical setting when it has been used for remission induction in relapsed AML prior to alloHSCT. 

### 2.4. Treatment of Relapsed AML with FLT3 Mutations

In case of AML relapse, with detection of either *FLT3-ITD* or *FLT3-TKD* mutations at primary diagnosis, confirmation of the *FLT3* mutational status is required, because clonal evolution can confer the loss of activating mutations at relapse [16,17]. After combined treatment with intensive chemotherapy and midostaurin, loss of activating *FLT3* mutations is observed in half of patients at AML relapse or disease progression [18].

Recent phase 3 clinical trials investigated the clinical outcome of AML patients at relapse following treatment with such *FLT3* tyrosine kinase inhibitors (TKI) as quizartinib or gilteritinib compared with intensive salvage chemotherapy regimens. Notably, quizartinib can effectively address *FLT3-ITD*, but not *FLT3-TKD* mutations, and the inhibition of *KIT* by its “off target” activity contribute to prolonged cytopenia [19]. The application of gilteritinib that is highly effective against both kinds of *FLT3* mutations has been shown to achieve second remission with less toxicity compared to intensive salvage chemotherapy regimens and currently offers the best choice of “bridging to transplant” in this challenging situation [20]. Moreover, gilteritinib is still effective in patients who were treated with midostaurin or sorafenib during AML first-line therapy [21].

### 2.5. FLT3 Mutations in AML Patients Not Eligible for Intensive Treatment

So far, no *FLT3*-directed TKI has been approved for first-line treatment of AML patients with activating *FLT3* mutations who are not eligible for intensive chemotherapy. The current standard of care for these patients is the combined treatment with hypomethylating agents (HMA) plus venetoclax [22]. A retrospective analysis of AML patients with activating *FLT3* mutations presented by Aldoss and co-workers revealed an excellent composite CR (CRc = CR/CRi) rate of 94% for treatment-naïve AML patients undergoing combined treatment with HMA plus venetoclax. Even in r/r AML patients harboring either *FLT3-ITD* or *FLT3-TKD* mutations HMA plus venetoclax was able to achieve a CRc rate of 42% [23].

Preclinical studies could demonstrate synergistic effects between venetoclax and quizartinib in murine AML models [24]. Recently, first clinical data of triplet therapy consisting of venetoclax, gilteritinib, and decitabine have been presented. An analysis of 12 newly diagnosed and 13 patients with r/r AML supporting this combination treatment as feasible and highly effective for AML patients harboring activating *FLT3* mutations [25]. Similar results have been obtained from a still recruiting clinical trial investigating the combination of venetoclax, quizartinib, and decitabine [26].

Thus, current treatment approaches for AML patients with activating *FLT3* mutations might provide not only feasible but also highly effective targeted therapy combinations that have the potential of remission induction also in younger patients who are eligible for alloHSCT. 

## 3. Inhibitors of IDH1 and IDH2

Presence of isocitrate dehydrogenase (IDH) mutations in AML blasts was described shortly after its discovery in glioblastoma [27,28]. The frequency of *IDH1* and *IDH2* mutations in AML is approximately 8% and 12%, respectively [29]. The impact of those epigenetic mutations is an overproduction of the oncometabolite 2-hydroxyglutarate (2-HG) [30]. Both DNA methylation as well as histone modification is inhibited by 2-HG [31]. *IDH1* and *IDH2* heterozygous missense mutations occur at conserved single arginine residues in the enzyme active site [32]. Specifically, the R132 locus of *IDH1* and R140 or R172 in *IDH2* are affected, respectively. The neomorphic enzyme activity acquired by mutated *IDH* (mIDH) and consequently aberrant 2-HG production leading to an increase in DNA hypermethylation and a myeloid differentiation block [33]. 

Impact of *IDH* mutations on outcome is still conflicting and co-mutations, such as *NPM1* or *DNMT3A*, appear to be important [34]. Mutated *IDH2* (R172K) has been associated with a favorable prognosis [35]. Larger studies are warranted to shed light in the inconsistencies between studies addressing the prognostic impact of *IDH1/2* mutations. 

Two *IDH*-inhibitors, enasidenib (IDHIFA, AG-221) for *mIDH2* and ivosidenib (TIBSOSOV, AG-120) for *mIDH1* were recently approved by the FDA for r/r AML based on two single arm studies [36,37]. Ivosidenib also received FDA approval for newly diagnosed AML in patients not eligible for intensive therapy [38]. In contrast, none of the *IDH* inhibitors has yet received European approval. 

Enasidenib is a selective allosteric inhibitor of *mIDH2* enzyme that inhibits the conversion of alpha-ketoglutarate (α-KG) to 2-HG thereby achieving a significant reduction in plasma 2-HG levels [39]. Within the phase 1/2 clinical trial, which led to the approval of the drug, 214 patients with r/r AML were treated at the 100 mg enasidenib dose level daily [36]. Overall response rate (ORR) was 39% with a median duration of 5.6 months and CRc rate was 29% (CR 19.6%, CRi/CRp 9.3%). The median OS among all r/r AML patients was 8.8 months with a median OS for patients attaining a CR of 22.9 months [36]. 

Ivosidenib, a selective *mIDH1* inhibitor also shows strong 2-HG inhibition and reinstatement of myeloid differentiation [40]. FDA approval for AML was granted in face of the phase 1/2 trial, including 152 patients in the r/r AML cohort with a recommended dose of 500 mg daily [37]. The CRc rate was 30% with an ORR of 42% and a median duration of CRc lasting 8.2 months. The median OS was 8.8 months, and 18-month survival of 50% of those patients who achieved CRc. 

Based on the results of the original ivosidenib monotherapy trial by DiNardo and co-workers that included 33 patients with newly diagnosed/treatment naïve *mIDH1* AML the drug was also approved as first line monotherapy in patients ineligible for intensive therapy. Median age of these 33 patients was 77 years and 76% had secondary AML whereas most of them already received prior therapy with HMAs. CRc rate of 42.5% (CR rate of 30%) was attained with a median OS of 12.6 months [38]. 

Concerning adverse events of both *IDH1/2* inhibitors show good tolerability but clinicians should be aware of the development of differentiation syndrome (DS). This has been reported in about 12–15% of patients receiving *IDH* inhibitor monotherapy and most frequently results in non-specific syndromes, such as fever, dyspnea, hypotension, pulmonary infiltrates, and hypoxia [41].Treatment of choice in IDH-DS is discontinuation of the *IDH* inhibitor and application of steroids (dexamethasone 10 mg bid) [42].

Several mechanisms of resistance have been identified whereas primary and secondary causes are described. Co-occurring mutations in the RAS pathway, leading to both primary and secondary therapeutic resistance, and it has been demonstrated that AML patients harboring such mutations are less likely to achieve a response to IDH inhibition [43]. 

Newly acquired mutations within the receptor tyrosine kinase genes and mutations restoring 2-HG levels contribute to secondary resistance [44]. 

In conclusion, the development of those oral, small molecule *mIDH1/mIDH2* inhibitors represents an important cornerstone on the way to personalized treatment in AML patients. Since early relapses occur, clinical investigations are warranted to overcome mechanisms of resistance (e.g., by combination strategies with intensive chemotherapy, HMAs, venetoclax, or other targeted therapies). 

## 4. Epigenetic Treatment of AML 

As an important mechanism in different types of cancer, aberrant hypermethylation of specific promotor regions contributes to elementary alterations in gene function, and cell regulation [45]. Today, HMAs, such as 5-azacitidine (AZA) or decitabine (DEC), are acting as inhibitors of DNA methyltransferases (DNMT), and are widely implemented in standard care of older and fragile AML patients. Introduction of HMAs has significantly improved OS compared to conventional regimens, such as low dose cytarabine (LDAC) or more intensive chemotherapy. Due to its well tolerability and efficacy compared with conventional palliative chemotherapy, HMAs became the backbone as well as in first line treatment in elderly patients not eligible for intensive treatment and as a treatment option in r/r AML [46,47,48]. Nevertheless, survival rates are not satisfying and efforts in increasing HMA efficacy are ongoing. 

Currently, due to the pharmacological profile of AZA or DEC, respectively, treatment with HMAs requires subcutaneous or intravenous application over 5 to 7 days in 28-day cycles. In the phase III, multicenter and placebo-controlled QUAZAR AML-001 trial (median age 68 years, range 55–86) a novel oral formulation of AZA (CC-486) as maintenance therapy for patients with de novo AML in first remission after induction chemotherapy which are ineligible for subsequent alloHSCT has been investigated. In detail, a significant prolongation of OS (24.7 vs. 14.8 months for CC-486 and placebo, respectively) and PFS (10.2 vs. 4.8 months for CC-486 and placebo, respectively) with comparable adverse event profiles to injectable AZA could be demonstrated. AML relapse was observed in 60% of patients in the AZA group and in 77% in the placebo cohort, respectively [49]. 

For oral AZA, a more sustained epigenetic activity over the treatment course by a prolonged exposure time over 14 days is hypothesized. Notably, pharmacokinetic analysis demonstrated significant differences compared to parenteral AZA in terms of metabolization, while CC-486 is not considered as bioequivalent [50]. In the QUAZAR AML-001 trial, CC-486 compared to the placebo was also associated with a significantly reduced length and risk of hospitalization, which increases the patient’s quality of life and substantially safes costs [51]. FDA and EMA approvals were granted in September 2020 and June 2021, respectively, for treatment of AML in first remission as maintenance therapy following intensive induction chemotherapy who are not able to finish curative intended chemotherapy or undergo consolidation with alloHSCT. 

De Lima and co-workers investigated CC-486 in a phase II trial as maintenance treatment in the situation of CR following alloHSCT [52]. They could demonstrate low relapse rates, low disease progression, and low GvHD rates as well as a good tolerance to drug exposure. 

Furthermore, HMA in combination with donor lymphocyte infusions (DLI), in case of molecular relapse following alloHSCT, is a therapeutic option, which was investigated in the phase II RELAZA trial [53]. Another prospective phase II trial is investigating a potential additive effect of the immunomodulator lenalidomide as addition to AZA + DLI in setting of relapse of myelodysplastic syndrome (MDS) and AML with MDS related changes following alloHSCT. An interim analysis demonstrated a promising ORR of 68% and a median molecular relapse-free survival (RFS) of 183 days (range, 113–513) [54].

### The Role of Venetoclax in HMA-Based Treatment

Recently, the BCL-2 inhibitor venetoclax has received FDA and EMA approvals for previously untreated elderly and unfit patients in combination with HMA or LDAC representing a remarkable improvement in a hard-to-treat patient cohort [55,56]. The underlying data result from the open labeled, multicenter VIALE-A trial including 431 previously untreated AML patients (median age of 76 years) randomized in a 2:1 fashion into AZA/venetoclax and AZA/placebo, respectively. With a median follow-up of 20.5 months (range, 0.1 to 30.7 months), a significantly improved OS of 14.7 months for AZA/venetoclax compared to 9.6 months for AZA alone was achieved. Moreover, a higher CR rate (17.9% vs. 36.7%) to favor of the HMA plus venetoclax group could be shown [22]. An improvement was also seen presented in phase III VIALE-C trial when venetoclax was combined LDAC [57]. Both trials set a new standard in treatment of elderly AML patients, while the search for response predicting molecular signatures and subgroups is ongoing. In detail, *NPM1*-mutations are associated with excellent survival and response rates while *TP53* or *FLT3-ITD* mutations are predictors for resistance towards HMA plus venetoclax [58]. 

The use of venetoclax in various treatment scenarios is under intensive investigation. Application of HMA plus venetoclax in the situation of r/r AML has not been analyzed systematically in clinical trials so far. In a phase II study, including 32 patients with r/r AML who received monotherapy venetoclax, only a moderate ORR with 19% was seen [59]. However, published retrospective data for HMA combination with venetoclax are providing promising results in treatment of patients with r/r AML where suitable treatment options are rare [60,61,62,63,64].

HMA plus venetoclax is also considered in relapsed AML following alloHSCT. A retrospective analysis of 32 patients demonstrated satisfying response rates when HMA/venetoclax is applied as salvage treatment or early at molecular relapse [65,66]. Using prior to alloHSCT HMA/venetoclax has shown an excellent ORR of 68.8% in a small cohort of 32 patients (including 19 with r/r AML and 13 with de novo AML), providing a feasible strategy of remission induction [67]. 

Improvement of response by adding venetoclax to intensive induction chemotherapy regimens is subject of current research. Results of phase I and II trials for the combination of FLAG-IDA (fludarabine, cytarabine, idarubicin and G-CSF) with venetoclax show convincing results regarding efficacy (ORR 70–97%), deep response (96% of de novo AML achieved MRD negative CR) accompanied by an acceptable safety profile [68]. Additionally, phase I trials combining venetoclax with frontline 7 + 3 daunorubicin/cytarabine based induction treatment (NCT03709758) or a phase II trial evaluating venetoclax, cladribine, AZA and LDAC combination for previously untreated AML patients in frontline (NCT03586609) will further yield evidence.

Preclinical studies indicate synergistic effects between *FLT3* inhibitors plus venetoclax, which mainly seems to be caused by downregulation of *MCL-1* und *BCL*(*x*)*L* [24,69,70]. Early clinical studies subsequently show positive effects and an acceptable safety profile for the combinations of *FLT3* inhibitor plus HMA and *FLT3* inhibitor plus venetoclax with ORRs of 65–80% and 85%, respectively [71,72]. Several studies investigating *FLT3* inhibitors plus venetoclax combinations are ongoing: a phase I trial testing a combination of venetoclax and the second-generation TKI gilteritinib (NCT03625505) or a phase Ib/II trial for venetoclax and quizartinib, both in patients with *FLT3* mutated r/r AML (NCT03735875). A Phase Ib study is combining gemtuzumab ozogamicin with venetoclax in patients with *CD33* positive r/r AML (NCT04070768).

## 5. Mechanisms of Resistance towards HMA and Venetoclax

Despite high efficacy of HMA/venetoclax combinations, one-third of patients fail to respond. Several cellular mechanisms and molecules causing drug resistance have been identified; however, there is an urgent need of a deeper understanding to predict and overcome resistance [58]. The following section provides a detailed overview about mechanisms of resistance towards HMA and venetoclax therapy.

### 5.1. Hypomethylating Agents (HMAs) 

HMAs are acting as pyrimidine analogs of the nucleoside cytidine. Its incorporation into DNA leads to an irreversible link between the cytidine analogue and the DNA methyltransferase (DNMT), which causes inhibition of the latter. HMAs show differential cellular effects depending on its dosage. High dose levels result in short term cytotoxic effects by direct DNA damage through DNMT-DNA adducts while at lower dose hypomethylating and epigenetic effects lead to improved cell differentiation and tumor suppression [73,74,75,76].

A crucial step is the uptake of HMA into the cell through the human equilibrated nucleoside transporter-1 (hENT1) [77]. Elevated mRNA transcript levels of this receptor are associated with improved response rates to DEC while lower expression can contribute to primary resistance [78].

As HMAs are applicated as prodrugs, requirement of a stepwise phosphorylation to active cytosine derivatives by di- and triphosphatases is dependent on deoxycytidine kinases (DCK). Low DCK expression has been attributed to DEC resistance in AML cell lines [78,79]. Cytosine derivatives underlie a natural degradation mediated by cytidine deaminases (CDA). The expression level of CDA is known to be a predictor for HMA response and thus representing a potential target to improve sensitivity to HMAs [80]. Besides that complex field of HMA metabolism and processing, several studies evaluated the impact of molecular aberrations towards HMA. *TET2* mutations as well as alterations of *IDH1/2*, *SF3B1*, or *DNMT3A* represent positive predictors for response towards HMAs in MDS and AML [81,82,83,84]. On the other hand, mutations in *ASXL1*, *PTPN11*, *CDKN2A*, *ETV6*, and *FLT3-ITD* were associated with shorter OS and lower ORR [85,86]. In a large retrospective registry analysis of 311 patients, no significant differences between patients with TP53 wild type or mutations regarding response towards DEC could be detected. Therefore, the influence of TP53 mutations in this setting remains controversial [87]. 

### 5.2. Venetoclax 

The *BCL-2* protein family comprises multiple regulator proteins, which directly affect intrinsic apoptosis pathway at the mitochondrion. In detail, anti-apoptotic proteins like *BCL-2*, *MCL-1*, or *BCL*(*x*)*L* can bind and sequester the effectors *BAK* and *BAX* and prevent their function of oligomerization and induction of mitochondrial outer membrane permeabilization (MOMP). Consequently, subsequent release of cytochrome c and caspase 8 activation leading to cell death [88,89]. This reaction can be gained by the small BH3-only proteins (e.g., *BID*, *BAD*, *BIM*, *PUMA*, *NOXA*), representing competitive inhibitors of anti-apoptotic proteins leading to release and activation of the effectors *BAK* and *BAX* [90]. 

Usually, a strict balance between the anti- and pro-apoptotic effectors controls cell regulation. Venetoclax is mimicking BH3-only proteins through competitive binding of the anti-apoptotic protein *BCL-2,* which in turn leads to release of *BAK* and *BAX* subsequently inducing apoptosis. Overexpression of *BCL-2* in several lymphoid malignancies yielded to implementation of venetoclax in different disease entities, such as chronic lymphocytic leukemia. Myeloid blasts are not necessarily dependent on *BCL-2* expression and leukemogenesis is driven by different apoptosis suppressors like *MCL-1*, *BCL*(*x*)*L*, *BCL*(*w*) [91,92,93]. 

In the context of AML, a comprehensive experimental analysis of Zhang and colleagues revealed high expression levels of *BCL2A1* as a potential key mechanism for venetoclax resistance. Hereby, *BCL2A1* overexpression is described to be even more frequent than *BCL-2* overexpression and seems to be predominantly in transformed, secondary AML or in the subset of M4/M5 according to FAB (French-American-British) classification. Knockdown experiments of *BCL2A1* induced strong apoptosis in cell line models occurring almost exclusive in AML blasts, while sparing normal hematopoietic stem cells. This emphasizes *BCL2A1* as a potentially attractive and important target for AML therapy [94].

The observation of distinct regulation of cellular pathways in venetoclax resistant cells, e.g., activation of *MAPK* and *AKT*, underlies different molecular patterns mediating primary or secondary resistance [58,95]. In an extensive study inactivation or low levels of *TP53*, *BAX*, *BAK*, *NOXA* (*PMAIP1*), *TFDP1* have been identified as conferring resistance towards venetoclax [96]. Additionally, inactivation of the tumor suppressor *TP53* was mediating alterations in expression of *BCL-2* family members and thereby had an impact on mitochondrial homeostasis and cellular metabolism. An increase of mitochondrial stress turns cells into resistance towards different anti-cancer drugs like *FLT3* inhibitors—even besides venetoclax. This underlines the tight link of mitochondrial stress (e.g., through *TP53* mutations) and expression of anti-apoptotic proteins. 

Mutations in genes like *KRAS*, *PTPN11*, and *FLT3-ITD* are known to confer primary or secondary drug resistance. In *KRAS* mutated cell line models harboring an activating gain of function mutation *G12D*, consecutive overexpression of *BCL2A1* and *MCL-1* as well as reduced levels of *BCL-2* and *BAX* have been described, which is crucial for venetoclax resistance [94,96]. 

Protein tyrosine phosphatase non receptor type 11 (*PTPN11*) encodes for the non-receptor protein tyrosine phosphatase *SHP2* (SH2 containing protein tyrosine phosphatase-2), which positively mediates downstream signaling from receptor tyrosine kinases like *FLT3* or *KIT* [97]. Therefore, gain of function mutations (e.g., *E76K*, *A72D*) in the *PTPN11* gene convey activation of downstream pathways, such as *RAS*, *JAK-STAT,* and *MAPK*. Thereby effects of *MCL-1*, *pMCL-1*, and *BCL*(*x*)*L* are intensified and venetoclax sensitivity is reduced [98,99], especially the co-occurrence of *FLT3-ITD* and *SHP2* activates *STAT5* signaling, which strongly induces anti-apoptotic signaling (e.g., *MCL-1*) and promotes hematopoietic cell cycle progression and survival [100,101,102,103,104,105]. Similar mechanisms are known for *FLT3-ITD* mutations [58,106]. 

**Figure 1:**
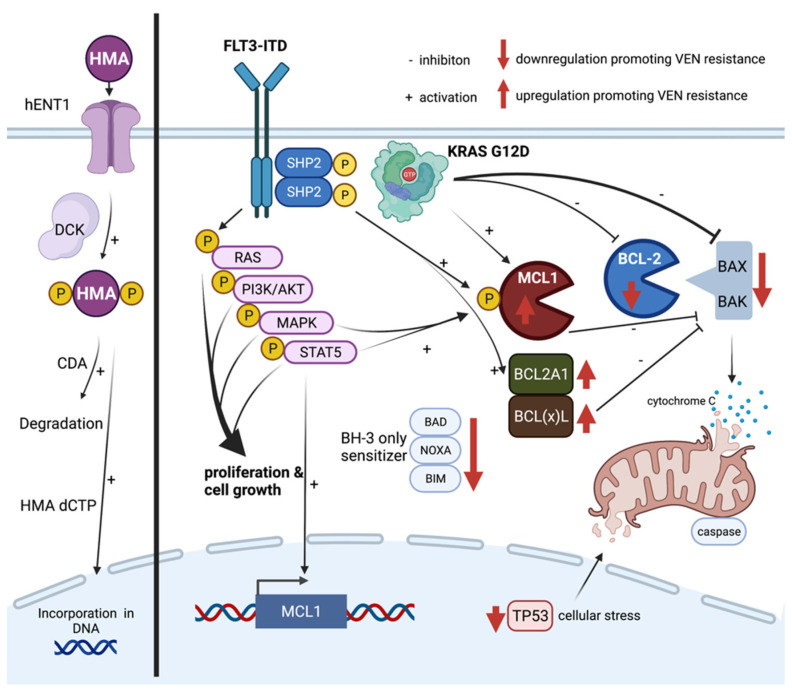
Mechanisms of resistance towards venetoclax. Left: uptake, processing, and incorporation of HMAs into the leukemic cell are illustrated. Cellular uptake of HMAs mainly depends on cell surface *hENT1* receptor expression. Phosphorylation to active cytosine di- and triphosphatases is mediated by *DCK*, whereas low expression and diminished activity contributes to decreased HMA effect. Augmented enzymatic degradation of dCTPs also contributes to degraded HMA effect. Right: regulation and interactions of proteins that are responsible for venetoclax resistance are shown. Balance between multiple *BCL-2* members within the concert of intrinsic apoptosis pathways is pivotal. Downregulation of *BCL-2* reduces venetoclax sensitivity as well as reduced activation of mitochondrial apoptosis effectors *BAX* and *BAK. KRAS* mutations mediate such gene expression alterations. Upregulation of important anti-apoptotic genes like *MCL-1*, *BCL2A1,* and *BCL*(*x*)*L* causes venetoclax resistance by binding and sequestering the effectors *BAX* and *BAK*. Activation and upregulation are driven by activating mutations of *FLT3-ITD*, *SHP2,* or *KRAS*. Especially consecutive *STAT5* signaling can directly upregulate *MCL-1* by activating an *MCL-1* promotor. *BH3*- only proteins acting as pro-apoptotic sensitizers bind pro-apoptotic members causing configuration changes and release of *BAX* and *BAK*. Mutations in *TP53* can reduce its function as tumor suppressor and confer alterations of expression of *BCL-2* family members. One consequence of *TP53* deletion is an increase of cellular stress, which is known to contribute resistance towards different cancer drugs. Created with BioRender.com. Abbreviations: HMA hypomethylating agent; hENT1 human equilibrated nucleoside transporter-1; DCK deoxycytidine kinase; CDA cytidine deaminases; dCTP deoxycytidine triphosphate; FLT3-ITD FMS-like tyrosine kinase receptor III with internal tandem duplication; SHP2 SH2 containing protein tyrosine phosphatase-2; BCL-2 b-cell lymphoma 2; MOMP mitochondrial outer membrane permeabilization; KRAS Kirsten rat sarcoma virus; MCL-1 induced leukemia cell differentiation protein; BCL2A1 BCL-2 related protein A1; BCL(x)L b-cell lymphoma extra-large; STAT5 signal transducer and activator of transcription 5; TP53 tumor protein p53; VEN venetoclax.

Figure 1 demonstrates a selection of relevant cellular mechanisms contributing to venetoclax and HMA resistance. 

## 6. Potential Strategies to Overcome Venetoclax Resistance in AML

Based on the described mechanisms of venetoclax resistance, potential pharmacological strategies to overcome venetoclax failure are highlighted in this chapter. 

Cell metabolism and mitochondrial structures are known as important targets. In a genome-wide CRISPR/Cas9 screen in human AML cells, Chen and colleagues demonstrated the association of the depletion of genes necessary for mitochondrial organization and integrity with an increased sensitivity towards venetoclax. In detail, considering that expression of the mitochondrial structure protein *CLBP* (caseinolytic peptidase B protein homolog) is elevated in venetoclax resistant AML cells, knockdown of *CLBP* was leading to significant decrease of IC_50_ for venetoclax. Even in context of *TP53* depletion, knockdown of *CLBP* was able to rescue *TP53*-mediated venetoclax resistance highlighting the potential of targeting *CLBP* and other mitochondrial structures [107]. Pharmacologic inhibition of mitochondrial protein synthesis with antibiotics like doxycycline or tedizolid was capable to overcome resistance towards HMA plus venetoclax in vitro and in vivo [108]. 

HMA/venetoclax combination effectively and selectively targets cellular energy metabolism, especially oxidative phosphorylation (OXPHOS) in LSCs. This mechanism is hypothesized a major contributor to high clinical effectiveness and durable responses of this drug combination. However, through activation of fatty acid oxidation (FAO), LSCs can maintain or reinforce OXPHOS, which in turn decreases venetoclax sensitivity. Thus, targeting and suppression of FAO might contribute to conserve HMA/venetoclax properties [109].

Current pharmacological strategies to overcome resistance towards *BCL-2* inhibition mainly rely on targeting *MCL-1* [110]. Overexpression of *MCL-1* prior to or subsequent *BCL-2* inhibition requires adequate *MCL-1* inhibition to induce effective apoptosis. Thus, abrogated binding of apoptosis sensitizers *BIM*, *BAD*, *NOXA*, *BIM*, and *PUMA* on *MCL-1* has been identified to initiate apoptosis [111]. Several *MCL-1* inhibitors such as A1012477 and S63845 are under preclinical and clinical investigation demonstrating high synergistic efficiency with concurrent *BCL-2* inhibition [112,113]. A phase I trial on r/r AML investigating that combination strategy has been initiated (NCT03672695). Another novel *MCl-1* inhibitor VU661013 provided additional evidence for rescuing venetoclax resistance in patient-derived xenografts [114]. For the *MCL-1* inhibitor AZD5991 a phase I trial in combination with venetoclax in r/r hematological malignancies has been launched recently (NCT03218683). Detailed knowledge about the applicability and safety profile as well the role of *MCL-1* in physiological, non-hematologic tissues should be acquired in further clinical trials [114,115].

In the presence of *FLT3-ITD* or *PTPN11* mutations, consecutive *MCL-1* and *BCL*(*x*)*L* activation causes reduced venetoclax sensitivity. Thus, addressing *MCL-1* in *FLT3-ITD* mutated cells seems to be of particular interest to improve drug sensitivity [106,116] Current efforts in combining venetoclax with FLT3 inhibitors to successfully address *FLT3-ITD* induced resistance are described above. In *PTPN11* mutations, ABT-263 (navitoclax) and ABT-737 as dual inhibitors of *BCL-2* and *BCL*(*x*)*L* and the *MCL-1* inhibitor AZD5991 were able to overcome venetoclax resistance. An important side effect of dual inhibitors was severe thrombocytopenia leading to discontinuation of early clinical trials [94,117,118,119]. 

Cyclin dependent kinase 9 (CDK9) has emerged as a potential target in cancer [120]. Bogenberger and colleagues could demonstrate in vivo and in vitro significant synergistical effects between the *CDK9* inhibitor alvocidib and venetoclax through affecting the balance of intrinsic apoptosis effectors, such as downregulation of *MCl-1* and upregulation of *BIM* or *NOXA* [121]. A comparable approach was pursued by Han et al. by applying cobimetinib in vivo and in vitro in order to target and inhibit the *MAPK* pathway, representing another key downstream pathway that upregulates *MCL-1* expression. Combination of cobimetinib with venetoclax was synergistic in 7 out 11 investigated cell line models including those that were resistant towards single agent treatment [95]. 

As described previously, the *MDM2* antagonist idasanutlin can lead to effective reconstitution of *TP53* thereby enhancing mitochondrial apoptosis. Several preclinical and early clinical phase I/II studies investigating the combination of idasanutlin with venetoclax, in vivo and in vitro. The superior effect compared to single agent treatment might partially be explained by enhanced *MCL-1* inhibition [122].

## 7. Inhibition of Hedgehog, Menin, or MDM2

### 7.1. Glasdegib

The balance between proliferation and differentiation of stem cells is minutely regulated and several critical pathways including the sonic hedgehog pathway (SHH) are involved in these processes. The SHH pathway is characterized by a high complexity and its activation confers cell cycle entrance and inhibition of apoptosis. The transmembrane protein *SMO* plays a key role in SHH pathway activation and its functional state is predominantly affected by the interaction of SHH ligands and the *SMO* suppressors *PTCH-1* and *PTCH-2* [123].

LSCs in patients with r/r AML are characterized by overexpression of SHH pathway proteins (e.g., *GLI1*) being associated with resistance to chemotherapy and inferior OS. Furthermore, it has been shown that sensitivity towards either chemotherapy or HMAs like AZA is enhanced by concomitant treatment with *SMO* inhibitors [124,125].

The small-molecule *SMO* inhibitor glasdegib is characterized by a shorter half-life compared to vismodegib or erismodegib resulting in a lower toxicity. Glasdegib has been improved for first line treatment of elderly AML patients who are not eligible for intensive chemotherapy, and is combined with LDAC. The underlying clinical trial could demonstrate a superior OS of 8.8 months for those AML patients who received combination therapy with LDAC and glasdegib. In contrast, AML treatment with LDAC alone resulted in an OS of only 4.9 months [126].

The current impact of this approved combination treatment for elderly and therapy-naïve AML patients’ needs to be interpreted with caution in consideration of the clinically relevant improvement demonstrated for the combination of AZA plus venetoclax resulting in a median OS of more than 14 months. Combined treatment with LDAC and glasdegib appears promising for those patients with anteceding epigenetic therapy (e.g., AML following MDS). Current clinical trials address the question of other combinations with glasdegib including HMA or venetoclax.

### 7.2. Menin

While targeted therapies, such as *FLT3* inhibitors or the *BCL-2* inhibitor venetoclax, are either directly addressing a constitutively active receptor tyrosine kinase identified by mutational analysis, or inhibiting an important anti-apoptotic protein in defined clinical setting, the biology of AML with MLL- (*KMT2A*) rearrangement (*KMT2Ar*) is almost unique and therapeutic strategies are more complex [127].

AML with *KMT2Ar* can be diagnosed in about 5% of adult AML patients and is associated with higher rates of treatment failure or AML relapse. Therefore, those patients are considered as poor prognosis and alloHSCT is recommended in first remission. In addition, approximately 5% of patients harbor partial tandem duplications within the *KMT2A* gene (*KMT2A-PTD*) demonstrating a similar biology as described for *KMT2Ar*-dependent AML as described below [128,129].

The multifunctional scaffold protein menin is critically involved in the regulation of gene expression in a tissue-specific manner. Menin directly interacts with transcription factors, chromosomal regulators, and gene promotors resulting in enhanced gene expression of regulated target genes. The large protein *KMT2A* as well as menin can induce expression of homeobox (HOX) genes representing the master regulators guarding self-renewal and differentiation of hematopoietic stem cells (HSCs). In detail, the histone H3 lysine 4 methyltransferase *KMT2A* exerts its multiple functions by epigenetic mechanisms. In case of *KMT2Ar* fusion proteins, such co-factors as *MEIS1* contribute to an aberrant gene expression program resulting in AML development, e.g., by induction of a differentiation block [130].

Preliminary clinical data exist for two menin inhibitors recently evaluated in AML patients with either *KMT2Ar* or *NPM1mut* in phase 2 expansion cohorts. The KOMET-001 trial investigated the menin inhibitor KO-539 in adult AML patients presenting first results at the Annual meeting of the American Society of Hematology (ASH) in 2020. The well-tolerated KO-539 showed no relevant interaction with clinically important CYP3A4 inhibitor and 8 of 12 patients enrolled into this trial were evaluable. Beside a patient with *KMT2Ar* developing tumor lysis syndrome at lowest KO-539 dose level, clinical activity could also be demonstrated in *NPM1mut* patients including one patient achieving MRD-negative CR after being refractory to conventional chemotherapy [131]. The AUGMENT-101 trial evaluating the menin inhibitor SNDX-5613 shows encouraging first results with an ORR of 54% including MRD negativity in about two-thirds of patients enrolled. A patient harboring both *KMT2Ar* and *FLT3-ITD* previously demonstrating resistance to chemotherapy and gilteritinib achieved cytogenetic CR with SNDX-5613 single treatment [132].

Notably, the nuclear chaperone protein *NPM1* accumulates in AML blasts in about 30% of patients due to *NPM1* frameshift mutations (*NPM1mut*). Subsequent disruption of physiological shuttling of the *NPM1* protein shares important biologically features with *KMT2Ar*-positive patients. In detail, *NPM1mut* can confer a similar gene expression pattern as described for *KMT2Ar* AML and maintenance of *HOX* gene expression in *NPM1mut* AML cells also depend on both wild-type *KMT2A* and menin. *NPM1mut* often co-occur with activating *FLT3* mutations leading to an impaired prognosis especially in case of a high ITD/wild-type ratio (*FLT3-ITD^high^*). In consideration that *MEIS1* can also induce *FLT3* gene expression, combined treatment of *NPM1mut/FLT3-ITD^high^* AML patients with menin inhibitors and *FLT3* inhibitors might represent a promising approach [127,133,134].

### 7.3. MDM2

The tumor suppressor gene *TP53* encoding the transcription factor *p53* plays a crucial role in many human cancers and mutations of *TP53* occur in a broad percentage of AML patients depending on the history of AML (e.g., t-AML following chemotherapy). Beside the presence of *TP53* mutations, the functional inactivation of *p53* is observed in most AML cases. MDM2 represents one of the most important negative regulators of *p53* and overexpression of MDM2 or inactivation of *ARF* (p14) are the main mechanisms of functional inactivation of *p53* in *TP53*-unmutated AML patients. Thus, inhibition of MDM2 reflects a promising therapeutic strategy in those AML patients lacking *TP53* mutations. MDM2 inhibitors can affect the *ARF-MDM-p53* axis enabling functional reactivation of unmutated *p53* [135,136].

Inhibitors of MDM2—so called “nutlins”—have already been investigated in clinical trials including AML patients with wild-type *TP53*. Regularly observed adverse events were mainly gastrointestinal and myelosuppression. The second-generation MDM2 inhibitor idasanutlin has been investigated in a large phase II clinical trial. In 76 r/r AML patients undergoing combined treatment with intermediate-dosed cytarabine and idasanutlin, a CRc rate of 29% was demonstrated with a median duration of response of 6.4 months. The MDM2 expression level could be elucidated as a promising biomarker for response prediction to MDM2 inhibitor treatment in this study. While CR was achieved in only one of 25 patients with *TP53* mutations, in the subgroup for *TP53* wild type AML patients CR could be achieved in 31% (22 of 71 patients). The initiated phase 3 study (MIRRORS) had to be stopped early due to failure of achieving study endpoints [137,138].

Beside combination treatment with either HMA or cytarabine, combined targeted therapy approaches are of special interest. Thus, combination with venetoclax has the potential to delay development of resistance to MDM2 inhibitors that is regularly observed by the emergence of *TP53* mutations or selection of subclones harboring MDM2 mutations disrupting its interaction with *p53*. Two clinical trials investigating the combination of idasanutlin and venetoclax are ongoing and preliminary data are available for one of these studies. The phase 1 part of one of these trials could determine the RP2D for idasanutlin and venetoclax and demonstrated an overall CR rate of 29% [139].

The compound ALRN-6924 as a first-in-class dual inhibitor of MDM2 and MDM4 is able to overcome resistance to MDM2 inhibitors resulting from MDM4 overexpression. ALRN-6924 is currently investigated in early-phase clinical trials with r/r AML and MDS patients [140].

The MDM2 inhibitor AMG-232 is structurally different from nutlin derivatives and has recently been evaluated, but failed to achieve clinical activity at least as single treatment in a phase 1 dose-escalation study. In contrast, combined treatment of AMG-232 with the *MEK* inhibitor trametinib showed response in a few patients including one sustained CR. Current trial concepts consider combination therapy with DEC or chemotherapy [141].

## 8. Other Targeted Therapies of AML

Beyond the scope of immunotherapeutic strategies that we will discuss separately, there are several additional and promising approaches of AML targeted therapy. Addressing distinct signaling molecules like mutant *TP53* or *CDK9* may overcome resistance to conventional AML therapy or improve outcome of AML patients who are not eligible for intensive chemotherapy regimens.

Table 1 summarizes current treatment concepts of targeted therapies in AML and provides an overview of most recent advances in the development of new drugs in clinical trials.

## 9. Immunotherapy of AML

### 9.1. Gemtuzumab Ozogamicin

After a complex history, the *CD33*-directed antibody-drug conjugate gemtuzumab ozogamicin (GO) has been approved based on the results of the ALFA0701 study. This phase III clinical trial investigated the impact of additional GO treatment in patients with newly diagnosed AML undergoing induction chemotherapy, with the 7 + 3 regimen containing cytarabine 200 mg per square meter as continuous infusion (days 1 to 7) and daunorubicin, 60 mg per square meter, for three consecutive days (days 1 to 3). Consolidation chemotherapy also contained combination treatment with cytarabine and daunorubicin. Due to a high rate of adverse events (e.g., veno-occlusive disease, VOD) in previous studies, GO was administered at lower doses (3 mg per square meter at day 1 and day 4 with a maximum dose of 5 mg) [148].

The final results of the ALFA0701 trial confirmed a significantly improved event-free survival (EFS) for those patients treated with GO. The OS benefit was restricted to AML patients with favorable and intermediate subtype according to ELN classification. In detail, OS for both ELN subgroups was 45.6 months for the GO-arm compared to 26.9 months for the control group without achieving statistical significance (HR 0.730, 95% CI 0.489–1.089, *p* = 0.1216) [3,149].

Furthermore, a frequent single nucleotide polymorphism (SNP) of the *CD33* gene (rs12459419) has been associated with an improved EFS and OS in pediatric AML patients undergoing chemotherapy combined with GO. This polymorphism caused a truncated *CD33* protein, lacking part of the extracellular epitope recognized by GO. In contrast, the impact of this *CD33* SNP was not observed in adult AML patients receiving GO treatment within two large MRC trials. In addition, this analysis confirmed the lack of association of *CD33* expression level and response to GO treatment [150,151].

Several open questions, in terms of dosing and the clinical benefits of GO remain, including the impact of GO during consolidation chemotherapy on the survival of AML patients. Results of different clinical trials have been contradictory and several studies were not able to demonstrate a survival benefit for the addition of GO to consolidation chemotherapy [152,153].

In contrast, the recently published AMLSG-09-09 phase III trial investigated consolidation treatment with high-dose cytarabine and all-*trans* retinoic acid (ATRA), with or without GO in AML patients with *NPM1* mutations. A clinical benefit could be demonstrated for women, patients up to the age of 70 years and for patients without *FLT3-ITD* as demonstrated by EFS and CIR (cumulative incidence of relapse) subgroup analysis. Particularly, a companion study of the AMLSG-09-09 trial revealed a significantly enhanced reduction of *NPM1* MRD level in the GO group resulting in a significant decrease of CIR. Thus, the addition of GO during consolidation chemotherapy improves both the molecular response and the outcome of ELN favorable and intermediate risk AML patients [154,155]. 

### 9.2. BiTEs and Bispecific Antibodies

In contrast to antibody drug conjugates, such as GO, bispecific T cell engager (BiTE) molecules represent promising treatment approaches for AML patients. One of the most extensively studied BiTE blinatumomab being already approved for r/r and *CD19*-expressing acute lymphoblastic leukemia, demonstrates high remission rates including patients with MRD negativity [156,157].

Immunotherapy of AML can either consider neoantigens that arise from AML-specific mutations specific for the individual patient or addresses AML-associated antigens. Due to the high complexity and HLA-dependence of patient-specific neoepitopes that can be presented to the immune system after processing of neoantigens, this approach has not been implemented into clinical trials yet. In contrast, many AML-associated proteins are expressed on the cell surface or intracellularly provide the rationale for promising treatment strategies [158].

In general, toxicity (e.g., cytokine release syndrome (CRS) or tumor lysis syndrome)) of such immunotherapeutic approaches does not only result from the interaction between BiTE or antibody construct and the target cell (“on-target on tumor activity”). Side effects can arise from so-called “on-target off tumor toxicity” due to the expression of the target antigen on other cell types beyond the hematopoietic system. With respect to detection of treatment-relevant AML antigens on distinct blood cells, expression levels of target antigens vary between LSCs and HSCs or may be restricted to either of these compartments [159].

Currently, several clinical trials aim to investigate the impact of different BiTE molecules directed against AML surface proteins. Here, we will describe those BiTE strategies that have already been published or are characterized by relevant preliminary data. A common challenge of many BiTE approaches is their short half-life resulting in the necessity of continuous infusion of the drug as known for blinatumomab. Thus, a recent strategy is the development of half-life extended BiTE molecules that allow a short application of the drug by reducing its renal clearance. In the following, we give an overview about currently investigated BiTE strategies in AML patients that are also summarized in Table 2.

### 9.3. CD33

The most comprehensively investigated *CD33*-directed BiTE compounds are AMG 330 and the half-life-extended AMG 673. Results of a phase I AMG 330 trial including 55 r/r AML patients analyzed several dose steps of AMG 330 while AMG 330 needs to be dosed by continuous infusion. In detail, CRS of at least grade 3 was observed in 13% of patients and severity of CRS was associated with both AMG 330 dose-level and disease burden. CRc was documented in five patients, including one patient achieving CRmol [160].

The half-life extended *CD33xCD3* BiTE AMG 673 is administered by short infusion and it has been investigated in r/r AML. In detail, 30 patients with r/r AML were treated in ten dose-dependent cohorts and received between one and six cycles with AMG 673 demonstrating CRS in 50% (15 of 30) patients including 13% of patients with CRS grade ≥ 3. A total of 27 patients were evaluable for response assessment whereas a reduction in AML blasts of at least 50% could be demonstrated in 6 patients. In addition, one patient allocated to a higher AMG 673 dose level achieved CRi [161]. 

Further immunotherapeutic strategies addressing *CD33* on AML cells include the bispecific antibody GEM 333, the tandem diabody AMV564 and the *CD33xCD3* bispecific antibody JNJ-67571244, respectively, and are currently investigated in phase I clinical trials.

### 9.4. CD123

The high expression of *CD123* on LSCs as compared to HSCs appears to be very promising for the treatment of AML patients.

First results of a phase I clinical trial analyzing flotetuzumab first presented at the ASH meeting 2020 have been published recently including a total of 88 patients r/r AML patients. Patients with primary refractory AML or early relapse within 6 months were eligible for this study. In 30 patients fulfilling these criteria and treated with the recommended phase 2 dose, the observed CRc rate was 30.0%. Flotetuzumab was well tolerated including only 1 of 30 (3.3%) patient presenting a CRS ≥ grade 3 [162].

The bispecific antibody vibecotamab (XmAb14045) has been investigated in a phase I clinical trial including 106 patients while most patients had r/r AML. Vibecotamab was evaluated at three dose levels and applicated weekly in a 28-day schedule. While no tumor lysis syndrome was observed, CRS was documented in 62 of 106 patients and almost completely restricted to grade 1 or 2. In 51 patients receiving at least 0.75 µg/kg body weight, ORR was 14% (7 patients), including 5 patients with CRc. A low AML burden and distinct T cell subsets have been elucidated as potential biomarkers while there was no association with the *CD123* expression level [163].

### 9.5. FLT3 and CLL-1

The receptor tyrosine kinase *FLT3* (*CD135*) is not only constitutively activated in about 30% of AML patients, but also regularly expressed on bulk AML cells and LSCs as compared to HSCs. The BiTE construct AMG 427 is currently investigated in patients with r/r AML in a phase 1 clinical trial [164].

Considering that *FLT3-ITD* (approximately 25% of AML patients) is mainly bound to the endoplasmic reticulum and Golgi apparatus, it is a matter of debate whether a lower surface expression of *FLT3-ITD* might be associated with a reduced efficacy of treatment strategies addressing *FLT3* in those AML patients harboring *FLT3-ITD* [165].

The surface protein *CLL-1* (*CLEC12A*) is part of the C-type lectin superfamily and cannot only be detected on several cell types of innate immunity, but also on LSCs in AML patients. Since *CLL-1* is not expressed on HSCs, it represents a promising target not only for antibody-based immunotherapeutic approaches. The bi-specific antibody MCLA-117 is evaluated in a phase 1 clinical trial including r/r AML patients. Preliminary results analyzing 50 patients revealed CRS and pyrexia as the most common adverse events while grade ≥ 3 adverse events were rare. Response assessment including all allocated dose levels of MCLA-117 demonstrates 26 evaluable patients with blast reduction of at least 50% in four patients [166].

### 9.6. TIM-3-Directed Treatment

In contrast to HSCs, the expression of the T cell immunoglobulin and mucin protein 3 (*TIM-3*) can be detected on LSCs; therefore, it appears as a promising target molecule for AML treatment. *TIM-3* also represents a surface protein expressed on several subsets of immune cells with pleiotropic functions (comprehensively reviewed by Wang and colleagues). Briefly, *TIM-3* can be detected not only on CD4^+^ or CD8^+^ T cells, but also on natural killer (NK) cells and macrophages. Several ligands (e.g., *Gal-9* or *CEACAM1*) are able to bind *TIM-3* with distinct functions dependent on the cellular context. Its interaction with *TIM-3* actively regulates the functional state of such important cells as T cells or NK cells that can contribute to immunotolerance against AML cells [167].

The expression of *TIM-3* on LSCs in most AML patients is the rationale for targeting *TIM-3* directly on AML cells. An autocrine loop in LSCs secreting *Gal-9* can contribute to AML maintenance. This mechanism of self-renewal has been attributed to constitutive activation of the *NF-kB* pathway and to co-activation of beta-catenin in LSCs. Due to its dual function, addressing *TIM-3* is highly promising for treatment of high-risk MDS and AML patients [168,169,170].

The monoclonal antibody sabatolimab (MBG453) represents one of the best-investigated treatment approaches directed against *TIM-3* in AML and high-risk MDS patients. Sabatolimab has been studied in both first-line treatment and in r/r AML patients by combination with either the checkpoint inhibitor (PDR001, see below) or with HMAs. Recently published results of the phase I clinical trial (NCT03066648) could demonstrate an ORR of 41.2% with a 6-months duration of response (DOR) rate of 85.1% for the combination of sabatolimab and HMA in patients with newly diagnosed AML. Sabatolimab was well tolerated in patients with AML, MDS and CMML [171].

### 9.7. Checkpoint Inhibitors

Antibody-based strategies aiming at the activation of T cells have been approved for several solid tumors and contribute to an improved survival of patients, e.g., for lung cancer or melanoma. In AML, several ongoing clinical trials are investigating different treatment strategies considering classical checkpoint inhibitors, such as pembrolizumab, nivolumab, or a combination of nivolumab and ipilimumab. There is a broad spectrum of clinical settings being addressed by current clinical trials with checkpoint inhibitors ranging from AML patients with r/r AML to those who are in CR but characterized by a high risk of AML relapse including e.g., following alloHSCT [172,173].

Beside treatment approaches containing only one or two checkpoint inhibitors, several clinical trials are focusing on combination therapy with either conventional chemotherapy, epigenetic treatment with HMAs, or targeted therapy (e.g., venetoclax). One rationale of combination of conventional chemotherapy (e.g., AML induction chemotherapy) with checkpoint inhibitors is the observation of an enhanced susceptibility towards cytotoxic T cells following treatment with cytarabine. In contrast, HMA treatment can up-regulate PD-1 expression on T cells suggesting a potential clinical benefit for the combination with checkpoint inhibitors [174,175].

Table 3 summarizes current clinical trials investigating treatment concepts containing checkpoint inhibitors in combination with already approved therapies for AML patients. Thus far, no checkpoint inhibitor has been approved for treatment of AML.

### 9.8. CAR-T Cell Approaches in AML

The development of CAR-T cell treatment has significantly improved our therapeutic armamentarium for pretreated patients with diffuse large B cell lymphoma (DLBCL), mantle cell lymphoma (MCL), or acute B-lymphoblastic leukemia, and will also be implemented in treatment algorithms for follicular lymphoma and multiple myeloma [176].

A major challenge of CAR-T cell approaches in AML patients is the expression of several target proteins on healthy hematopoiesis resulting in prolonged cytopenia being associated with an even more increased risk of severe infectious complications or the necessity of subsequent alloHSCT. Another important biological aspect concerning CAR-T cell therapy in AML is the potentially reduced T cell number and function following conventional chemotherapy.

Current clinical trials with CAR-T cells in AML patients address *CD33*, *CD123*, *CLL-1*, *CD44v6*, and *NKG2D* as target molecules expressed on AML, while conditioning therapy prior to CAR-T cell application is similar to lymphodepletion regularly administered in lymphoma patients [159].

Efforts are ongoing to improve the specificity of CAR-T cells with respect to its “on-target but off-tumor” effects on HSCs and to reduce the toxicity profile (e.g., the severity of CRS). Two promising approaches to achieve those goals consider bispecific CAR-T cells. In detail, compound CAR (cCAR)-T cells contain two independent and functionally active CAR molecules (e.g., *CLL-1* and *CD33*). Preliminary results of the corresponding clinical trial describe MRD negative CR following cCAR-T cell treatment in r/r AML patients [177,178].

Another concept considering two target domains (*CD13* and *TIM-3*) being recognized by bispecific and split CAR (BissCAR) T cells has been developed by He and co-workers. This CAR-T cell approach has a high potential to enhance the specificity by reducing its toxicity against HSCs [179]. 

### 9.9. Novel Promising Targets: CD47 and CD70

In addition to the activation of T cells by BiTE molecules or bispecific antibodies, two important treatment strategies aiming at the activation of innate immunity against AML cells are reported. Both approaches have already been investigated in early clinical trials with promising results.

#### 9.9.1. CD47

The integrin-associated transmembrane protein *CD47* is widely expressed on AML cells and can suppress macrophages preventing leukemia cells from phagocytic elimination. The expression level of CD47 is associated with the clinical outcome of AML patients attributed to the unfavorable prognostic subgroup and characterized by an inferior EFS and OS [180,181].

The *CD47-SIRPalpha* axis that is also known as the “do not eat me” signaling can be disrupted by the first-in-class monoclonal antibody magrolimab (Hu5F9-G4). In a recently published clinical trial, 52 therapy-naïve AML patients with adverse prognostic markers, including patients with *TP53* mutations underwent magrolimab treatment in combination with AZA. Here, a high CRc rate of 50% including half of these patients with MRD negativity could be achieved in this study cohort resulting in a median OS of about 13 months. In patients with high-risk MDS, a phase 3 study is ongoing investigating the clinical impact of additional treatment with magrolimab [182,183].

Furthermore, this immunotherapeutic approach has the potential to eliminate LSC effectively. Thus, this special checkpoint inhibitor targeting the *CD47-SIRPalpha* axis is considered as a promising treatment approach not only for AML patient.

#### 9.9.2. CD70

The surface protein *CD70* represents an important ligand of the *TNF* superfamily receptor *CD27,* and can be detected on most AML blasts, while both surface molecules are not detectable in healthy bone marrow cells. The *CD70-CD27* axis can promote AML cell growth and contributes to the blockage of cell differentiation in AML. The soluble form of *CD27* (*sCD27*) can be detected in sera of AML patients displaying a negative prognostic marker in case of higher *sCD27* levels. The expression of *CD70* on LSCs in AML patients supports the hypothesis that the interaction between *CD70* and *CD27* plays a key role in LSC maintenance [184].

*CD70* can be targeted by the monoclonal antibody cusatuzumab that is characterized by an enhanced activity in terms of antibody-dependent cellular cytotoxicity (ADCC). In a recently published phase I study, cusatuzumab has been evaluated in a dose escalation cohort of 12 elderly treatment-naïve AML patients while all patients received a single dose of cusatuzumab followed by AZA treatment. The combination therapy of AZA and cusatuzumab was well tolerated at all dose levels. Importantly, 8 of 12 patients enrolled into this study achieved CRc including 4 patients with MRD negativity. In addition, the authors can demonstrate a significant reduction of LSCs following treatment with the *CD70*-targeting antibody cusatuzumab [185].

In consideration of the vulnerability of LSCs towards cusatuzumab, *CD70* represents an attractive target molecule for the improvement of AML therapy. Besides testing further treatment combinations (e.g., cusatuzumab plus AZA and venetoclax), *CD70*-directed CAR-T cell approaches are under development [186,187]. 

## 10. Effective Targeting of Leukemic Stem Cells

LSCs play a pivotal role in AML development, response to treatment and are responsible for the evolution of AML relapse following persistence of detectable MRD. LSCs can be characterized by a distinct immune phenotype within the *CD34+CD38*- AML fraction regularly expressing such important surface proteins, such as *CD123*, *TIM-3*, *CLL-1*, *CD70*, *CD44v6*, or *GPR56*. Some of these proteins are exclusively expressed on LSCs or display a higher expression level as compared to normal *CD34+CD38-* HSCs representing potential targets for immunotherapy approaches (e.g., BiTEs or CAR-T cells) in AML treatment [188].

The eradication of residual AML cells by addressing persistent LSCs by immunotherapy is also under investigation by means of reactivation of AML-directed T cells implementing checkpoint inhibitors in different clinical settings as described above. The expression of *TIM-3* on both LSCs and on a broad range of immune cells (e.g., T cells) makes *TIM-3* an attractive target for AML therapy [167].

Importantly, similar to clonal heterogeneity in “bulk AML” potentially responsible for persistent MRD and treatment failure by clonal selection under conventional chemotherapy or following targeted therapy (e.g., breakthrough of FLT3-ITD-negative clones under midostaurin maintenance therapy) distinct LSC populations are hypothesized in AML patients. Such a diversity on the level of AML LSCs contributes to a high complexity of addressing LSCs effectively [189,190,191]. 

Transcriptome profiling of LSCs revealed a LSC signature that can be attributed to leukemia-initiating capacity and so-called “stemness”. Moreover, LSCs are predominantly regulated by epigenetic mechanisms rather than by driver mutations as detected in “bulk AML” cells resulting in a characteristic LSC expression signature resulting from a unique chromatin and epigenetic landscape. Together with a high plasticity and heterogeneity, LSCs can develop a dynamic resistance to chemotherapy or other treatment approaches [191,192].

Beside clonal selection of different AML clones or distinct LSC subtypes due to heterogeneous mutational patterns, in terms of clinically relevant molecular aberrations LSCs in AML are characterized by a series of unique molecular features. In detail, precise regulation of cell cycle entrance, differentiation or apoptosis is at least in part maintained by highly conserved signaling pathways (e.g., *SHH* pathway). Thus, implementing targeted therapies addressing such pathways can not only reduce resistance to chemotherapy but also contribute to a deeper remission and potential improvement of survival in AML patients [123,126].

In addition to highly conserved signaling pathways, the interaction between LSCs and bone marrow microenvironment plays a crucial role. In detail, mobilization of LSCs from the bone marrow niche, e.g., by disruption of *CXCR4* signaling represents a promising approach of “LSC priming” to enhance the susceptibility of LSCs to chemotherapy [193]. 

Furthermore, quiescence of LSCs is strictly regulated and increased expression of the transcription factor *FOXM1* has been demonstrated to be crucial for survival and self-renewal of LSCs in *KMT2Ar*-derived AML. Upregulation of *FOXM1* can directly activate the Wnt/beta-catenin signaling pathway preserving LSC quiescence and promoting their maintenance [194].

Another critical mechanism of LSC protection is represented by the integrated stress response (ISR) pathway responsible for balancing LSCs between apoptosis and survival under distinct critical conditions. Thus, the ISR pathway can rescue LSC within the hypoxic bone marrow niche by inhibition of reactive oxygen species (ROS) production. The *CD34+CD38-* compartment is also characterized by an increased activating transcription factor 4 (*ATF4*) expression indicating higher IRS pathway activity and potential target for AML therapy [195,196].

**Figure 2:**
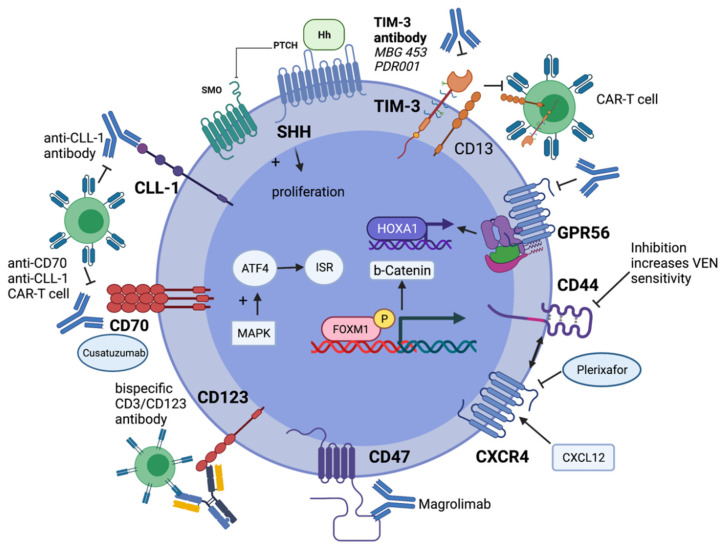
Current immunotherapeutic approaches targeting leukemic stem cells in AML patients. *TIM-3* is a membrane bound glycoprotein and immunoreceptor expressed on both LSCs and cytotoxic *CD4*^+^ and *CD8*^+^ T-cells. It can be addressed by monoclonal antibodies (MB453 or PDR001) and bispecific directed CAR-T cells against *CD13* and *TIM-3*. *GPR56*, a G-protein coupled transmembrane receptor, can be overexpressed in LSCs causing upregulation of leukemia driving transcription factor *HOXA1*. *CD44* is an adhesion molecule, which physically interacts with the transmembrane protein *CXCR4* and activated by *CXCL12,* which is crucial for anchorage of LSCs in the bone marrow niche. Inhibition of *CD44* overcomes resistance to *BCL-2* inhibitor venetoclax whereas *CXCR4* can directly be inhibited by plerixafor, leading to mobilization of LSCs from the bone marrow niche [197]. *CD47* is an integrin associated membrane protein and able to suppress macrophages and thus preventing eliminating of LSCs. The *CD47* directed monoclonal antibody magrolimab could disrupt the connection between *CD47* on LSC and *SIRPalpha* on macrophages. *CD123* as interleukin 3 receptor can be targeted by bispecific *CD3/CD123* antibodies to redirect T-cell to LSCs. *CD70* as a ligand of the TNF superfamily receptor *CD27* can be hindered from binding by monoclonal antibody cusatuzumab or *CD70* directed CAR-T cells. Same approaches are illustrated for *CLL-1* receptor. SHH and *MAPK* signaling is illustrated since they represent highly conserved pathways in LSCs which mainly contribute to maintenance and cell proliferation. *MAPK* is known to activate integrated stress response signaling which promotes cellular recovery and restore homeostasis. The transcription factor *FOXM1* is crucial for maintaining, survival, and renewing of LSCs, which is activated by b-*catenin/WNT* signaling. Created with BioRender.com. Abbreviations: TIM-3 T cell immunoglobulin and mucin-domain containing-3; AML acute myeloid leukemia; LSC leukemic stem cell; CAR-T chimeric antigen receptor t cell; GPR56 adhesion G protein–coupled receptor 56; HOXA1 Homeobox A1; CXCR4 C-X-C chemokine receptor type 4; CXCL12 C-X-C chemokine ligand type 12; BCL-2 b-cell lymphoma-2; TNF tumor necrosis factor; CLL-1 C-type lectin domain family 12 member A; SHH sonic hedgehog; MAPK Mitogen-activated protein; FOXM1 Forkhead Box Protein M1; VEN Venetoclax.

In addition to these promising targets that can be addressed in LSCs in a more specific manner, as compared to HSCs, several additional mechanisms of LSC maintenance have been investigated in pre-clinical models or even in early-phase clinical trials. Figure 2 summarizes the current concepts and important mechanisms that are in focus of future treatment strategies to eradicate LSCs in AML patients. 

## 11. Conclusions

Cytogenetic, especially molecular genetic analysis of AML cells, at diagnosis and at relapse, is indispensable for risk stratification with respect to alloHSCT consolidation treatment, and to implement target therapies up-front.

Treatment algorithms for elderly AML patients not eligible for intensive chemotherapy have been changed and the addition of venetoclax to epigenetic therapy has improved survival of elderly AML patients tremendously. Understanding the molecular mechanisms of primary or acquired resistance to venetoclax-based regimens in AML is of importance to improve second-line strategies for those patients not responding adequately to this treatment. Several candidates that are responsible for resistance to venetoclax were identified and molecularly defined strategies (e.g., inhibition of *MCL-1*) have the potential to overcome resistance in this clinical setting.

In consideration of the limited prognosis of elderly AML patients, future strategies might not only implement ex vivo characterization of primary AML cells. A comprehensive analysis considering resistance-conferring mutations (e.g., *PTPN11*) or the detection of individual phospho-proteome signatures able to reveal aberrant activation of signaling pathways able to mediate venetoclax resistance (e.g., MAPK activation) can contribute to improve future AML therapy.

While immunotherapy-based approaches have been implemented in the treatment of many entities, so far, gemtuzumab ozogamicin is the only antibody approved for AML therapy. Potential immunotherapeutic strategies either directly addressing AML surface proteins (e.g., *CD123*) or indirectly activating the innate immune system (e.g., macrophages by targeting *CD47*) are currently developed as single treatments or in combination with epigenetic therapy.

Future diagnostic approaches need to implement a much broader immunophenotype characterization in order to provide an optimal selection of immunotherapy for the individual AML patient.

The molecular and immunological characterization of LSCs provide substantial chances to improve response to AML therapy beyond controlling “bulk” AML cells and to achieve deeper remissions prior to alloHSCT to further increase survival of AML patients. 

Thus, both molecular and immunological characterization of AML cells should not be restricted to the whole population of AML cells, but instead focus on the prognostically relevant LSCs. Our vision for the future is a combined diagnostic approach, considering genetic and proteomic diagnostics that allow an integrated characterization for targeted therapy and the prediction of treatment resistance in AML.

Taken together, the armamentarium to treat AML is still growing, and the landscape of ongoing clinical trials contains promising new treatment approaches.

## Figures and Tables

**Table 1:** Targeted therapy approaches in r/r AML and corresponding clinical trials.

Target Molecule	Target Function	Rationale	Compounds in Development	Clinical Trials	Ref.
Polo-like kinase 1	G2->M entrance interaction with PI3K/mTOR	high expression of PLK1 in AML cells compared to CD34 progenitor cells	Onvansertib	NCT03303339: Phase 1b/2 study/45 pts. Onvansertib plus DEC or LDAC CR 9 pts. (20%), 4 pts with durable response of at least 9 months; 2 pts. proceeded to alloHSCT	[142]
TP53mutant	important tumor suppressor	poor prognosis of TP53mut AML restoration of TP53 function synergistic effect with AZA	APR-246 (Eprenetapopt)	NCT03072043: Phase 1b/2 study/55 pts., including 11 r/r AML pts. APR-246 plus AZA ORR 7 pts. (64%), CR 4 pts. (36%)	[143]
DOT1L	H3K79 methyltransferase	DOT1L plays a central role in leukemogenesis of AML with KMT2Ar AML	EPZ-5676 (Pinometostat)	Phase 1 study/43 pts. CR 2 pts., resolution of extramedullary AML 2 pts., signs of differentiation 9 pts.	[144]
BET	important epigenetic regulator	BRD4 overexpression in AML pivotal role in LSC transcriptional programs	Mivebresib (ABBV-075) pan-BET inhibitor	NCT02391480: Phase 1 study/44 pts. Mivebresib mono (MIV) or combination therapy with VEN (MIV-VEN) response MIV-VEN (30 pts.): CR 2 pts., PR 2 pts., MLFS 2 pts.	[145]
XPO1	nuclear transport protein	high expression of XPO1 in LSC modulation of protein shuttling	Selinexor (KPT-330)	NCT02093403: Phase 1 study/25 pts. Selinexor plus DEC ORR 40%, median PFS 5.9 months PFS responders 11.8 months	[146]
CDK9	regulation of RNA polymerase II activity	inhibition of transcription of genes involved in proliferation and survival (e.g., suppression of MCL-1)	Alvocidib (flavopiridol)	NCT03298984: Phase 1 study/32 pts. ND AML/Alvocidib -> 7 + 3 ORR 75%, CR 69%	[147]

Abbreviations: 7 + 3, induction chemotherapy with cytarabine and daunorubicin, alloHSCT, allogeneic hematopoietic stem cell transplantation; AZA, azacitidine; CR, complete remission; DEC, decitabine; LDAC, low-dose cytarabine; MLFS, morphological leukemia-free state; ND AML, newly diagnosed acute myeloid leukemia; ORR, overall response rate; PR, partial remission; r/r AML, relapsed or refractory acute myeloid leukemia; pts patients.

**Table 2:** Selected clinical phase I trials exploring immunotherapeutic approaches in r/r AML.

Target Molecule	Expression LSC/HSC	Drug	Mechanism of Action	Treatment Schedule	First Results	Clinical Trial	Ref.
CD33 (Siglec-3)	+/+	AMG330	CD33xCD3 BiTE	continuous infusion D1-D28 2 weeks off	55 pts. in 16 dose cohorts: AMG330-related AEs: 49/55 pts. (89%) CRS: 67%, grade ≥ 3: 13% Nausea: 11/55 (20%) Evaluable response in 42 pts. (17%) CRc: 7 pts.	NCT02520427	[160]
		AMG673	CD33xCD3 BiTE half-life- extended	Infusion @D1 and D5 every 14 days	30 pts. in 10 dose cohorts: CRS: 15/30 pts. (50%) TRSAE: 11/30 (37%) blast reduction > 50% in 6/27 pts.; CRi 1 pts.	NCT03224819	[161]
CD123 (IL3Rα)	++/(+)	Flotetuzumab (MGD006)	bispecific DART CD123xCD3	two schedules: 7-day CIV 4 days on/3 days off CIV	42 pts. dose-escalation/46 pts.@RP2D CRS: 81%, grade ≥ 3: 7% Nausea: 26% CRc @RP2D: 30%	NCT02152956	[162]
		Vibecotamab (XmAb14045)	Bispecific Antibody CD123xCD3	weekly infusion	CRS 62/106 pts. (58%) no TLS ORR@>0.75 µg/kg 7/51 pts. (14%) CRc 5/51 pts. (10%)	NCT02730312	[163]

Abbreviations: CIV, continuous intravenous infusion; CRS, cytokine release syndrome; TEAE, treatment-emerged adverse events, r/r AML, relapsed or refractory acute myeloid leukemia; TLS, tumor lysis syndrome; RP2D, recommended phase 2 dose; CRc, composite complete remission.

**Table 3:** Selection of ongoing clinical trials in AML with checkpoint inhibitors.

Clinical Trial	Molecular Stratification	Study Population	Checkpoint Inhibitor	Combination Therapy	Trial Phase
NCT03730012	*FLT3* mutation	r/r AML	Atezolizumab	Gilteritinib	Phase 1/2
NCT04044209	*IDH1* mutation	r/r AML	Nivolumab	Ivosidenib	Phase 2
NCT04277442	*TP53* mutation	1L AML	Nivolumab	Decitabine and Venetoclax	Phase 1
NCT03066648	none	r/r AML or 1L AML	PDR001	Sabatolimab (MBG453) or DEC	Randomized Phase 1
NCT03922477	none	r/r AML	Atezolizumab	Magrolimab (Hu5F9-G4)	Phase 1
NCT02890329	none	r/r AML	Ipilimumab	Decitabine	Phase 1
NCT02397720	none	r/r AML or 1L AML	Nivolumab ± Ipilimumab	AZA	Non-randomized Phase 1
NCT02464657	none	1L AML	Nivolumab	Idarubicin and Cytarabine	Phase 2

Abbreviations: r/r AML, relapsed or refractory acute myeloid leukemia; 1L AML, first-line treatment of acute myeloid leukemia; AZA, azacitidine; DEC, decitabine.

## References

[1] Nakao M., Yokota S., Iwai T., Kaneko H., Horiike S., Kashima K., Sonoda Y., Fujimoto T., Misawa S. (1996). Internal tandem duplication of the flt3 gene found in acute myeloid leukemia. Leukemia.

[2] Yamamoto Y., Kiyoi H., Nakano Y., Suzuki R., Kodera Y., Miyawaki S., Asou N., Kuriyama K., Yagasaki F., Shimazaki C. (2001). Activating mutation of D835 within the activation loop of FLT3 in human hematologic malignancies. Blood.

[3] Dohner H., Estey E., Grimwade D., Amadori S., Appelbaum F.R., Buchner T., Dombret H., Ebert B.L., Fenaux P., Larson R.A. (2017). Diagnosis and management of AML in adults: 2017 ELN recommendations from an international expert panel. Blood.

[4] O’Donnell M.R., Tallman M.S., Abboud C.N., Altman J.K., Appelbaum F.R., Arber D.A., Bhatt V., Bixby D., Blum W., Coutre S.E. (2017). Acute Myeloid Leukemia, Version 3.2017, NCCN Clinical Practice Guidelines in Oncology. J. Natl. Compr. Canc. Netw..

[5] Stone R.M., Mandrekar S.J., Sanford B.L., Laumann K., Geyer S., Bloomfield C.D., Thiede C., Prior T.W., Dohner K., Marcucci G. (2017). Midostaurin plus Chemotherapy for Acute Myeloid Leukemia with a FLT3 Mutation. N. Engl. J. Med..

[6] Voso M.T., Larson R.A., Jones D., Marcucci G., Prior T., Krauter J., Heuser M., Lavorgna S., Nomdedeu J., Geyer S.M. (2020). Midostaurin in patients with acute myeloid leukemia and FLT3-TKD mutations: A subanalysis from the RATIFY trial. Blood Adv...

[7] Bornhauser M., Illmer T., Schaich M., Soucek S., Ehninger G., Thiede C. (2007). Improved outcome after stem-cell transplantation in FLT3/ITD-positive AML. Blood.

[8] Oran B., Cortes J., Beitinjaneh A., Chen H.C., de Lima M., Patel K., Ravandi F., Wang X., Brandt M., Andersson B.S. (2016). Allogeneic Transplantation in First Remission Improves Outcomes Irrespective of FLT3-ITD Allelic Ratio in FLT3-ITD-Positive Acute Myelogenous Leukemia. Biol. Blood Marrow Transplant..

[9] Breitenbuecher F., Schnittger S., Grundler R., Markova B., Carius B., Brecht A., Duyster J., Haferlach T., Huber C., Fischer T. (2009). Identification of a novel type of ITD mutations located in nonjuxtamembrane domains of the FLT3 tyrosine kinase receptor. Blood.

[10] Kayser S., Schlenk R.F., Londono M.C., Breitenbuecher F., Wittke K., Du J., Groner S., Spath D., Krauter J., Ganser A. (2009). Insertion of FLT3 internal tandem duplication in the tyrosine kinase domain-1 is associated with resistance to chemotherapy and inferior outcome. Blood.

[11] Breitenbuecher F., Markova B., Kasper S., Carius B., Stauder T., Böhmer F.D., Masson K., Rönnstrand L., Huber C., Kindler T. (2009). A novel molecular mechanism of primary resistance to FLT3-kinase inhibitors in AML. Blood.

[12] Rucker F.G., Du L., Luck T.J., Benner A., Krzykalla J., Gathmann I., Voso M.T., Amadori S., Prior T.W., Brandwein J.M. (2021). Molecular landscape and prognostic impact of FLT3-ITD insertion site in acute myeloid leukemia: RATIFY study results. Leukemia.

[13] Burchert A., Bug G., Fritz L.V., Finke J., Stelljes M., Röllig C., Wollmer E., Wäsch R., Bornhäuser M., Berg T. (2020). Sorafenib Maintenance After Allogeneic Hematopoietic Stem Cell Transplantation for Acute Myeloid Leukemia With FLT3-Internal Tandem Duplication Mutation (SORMAIN). J. Clin. Oncol..

[14] Xuan L., Wang Y., Huang F., Fan Z., Xu Y., Sun J., Xu N., Deng L., Li X., Liang X. (2020). Sorafenib maintenance in patients with FLT3-ITD acute myeloid leukaemia undergoing allogeneic haematopoietic stem-cell transplantation: An open-label, multicentre, randomised phase 3 trial. Lancet Oncol..

[15] Mathew N.R., Baumgartner F., Braun L., O’Sullivan D., Thomas S., Waterhouse M., Müller T.A., Hanke K., Taromi S., Apostolova P. (2018). Sorafenib promotes graft-versus-leukemia activity in mice and humans through IL-15 production in FLT3-ITD-mutant leukemia cells. Nat. Med..

[16] Shih L.Y., Huang C.F., Wu J.H., Lin T.L., Dunn P., Wang P.N., Kuo M.C., Lai C.L., Hsu H.C. (2002). Internal tandem duplication of FLT3 in relapsed acute myeloid leukemia: A comparative analysis of bone marrow samples from 108 adult patients at diagnosis and relapse. Blood.

[17] Kottaridis P.D., Gale R.E., Langabeer S.E., Frew M.E., Bowen D.T., Linch D.C. (2002). Studies of FLT3 mutations in paired presentation and relapse samples from patients with acute myeloid leukemia: Implications for the role of FLT3 mutations in leukemogenesis, minimal residual disease detection, and possible therapy with FLT3 inhibitors. Blood.

[18] Schmalbrock L.K., Dolnik A., Cocciardi S., Sträng E., Theis F., Jahn N., Panina E., Blätte T.J., Herzig J., Skambraks S. (2021). Clonal evolution of acute myeloid leukemia with FLT3-ITD mutation under treatment with midostaurin. Blood.

[19] Cortes J.E., Khaled S., Martinelli G., Perl A.E., Ganguly S., Russell N., Kramer A., Dombret H., Hogge D., Jonas B.A. (2019). Quizartinib versus salvage chemotherapy in relapsed or refractory FLT3-ITD acute myeloid leukaemia (QuANTUM-R): A multicentre, randomised, controlled, open-label, phase 3 trial. Lancet Oncol..

[20] Perl A.E., Martinelli G., Cortes J.E., Neubauer A., Berman E., Paolini S., Montesinos P., Baer M.R., Larson R.A., Ustun C. (2019). Gilteritinib or Chemotherapy for Relapsed or Refractory FLT3-Mutated AML. N. Engl. J. Med..

[21] Perl A.E., Altman J.K., Hosono N., Montesinos P., Podoltsev N.A., Martinelli G., Smith C.C., Levis M., Röllig C., Groß-Langenhoff M. (2020). Clinical Outcomes in Patients with Relapsed/Refractory Acute Myeloid Leukemia Treated with Gilteritinib Who Received Prior Midostaurin or Sorafenib. Blood.

[22] DiNardo C.D., Jonas B.A., Pullarkat V., Thirman M.J., Garcia J.S., Wei A.H., Konopleva M., Döhner H., Letai A., Fenaux P. (2020). Azacitidine and Venetoclax in Previously Untreated Acute Myeloid Leukemia. N. Engl. J. Med..

[23] Aldoss I., Zhang J., Mei M., Al Malki M.M., Arslan S., Ngo D., Aribi A., Ali H., Sandhu K., Salhotra A. (2020). Venetoclax and hypomethylating agents in FLT3-mutated acute myeloid leukemia. Am. J. Hematol..

[24] Mali R.S., Zhang Q., DeFilippis R., Cavazos A., Kuruvilla V.M., Raman J., Mody V., Choo E.F., Dail M., Shah N.P. (2020). Venetoclax combines synergistically with FLT3 inhibition to effectively target leukemic cells in FLT3-ITD+ acute myeloid leukemia models. Haematologica.

[25] Maiti A., DiNardo C.D., Daver N.G., Rausch C.R., Ravandi F., Kadia T.M., Pemmaraju N., Borthakur G., Bose P., Issa G.C. (2021). Triplet therapy with venetoclax, FLT3 inhibitor and decitabine for FLT3-mutated acute myeloid leukemia. Blood Cancer J..

[26] Yilmaz M., Kantarjian H.M., Muftuoglu M., Kadia T.M., Konopleva M., Borthakur G., Dinardo C.D., Pemmaraju N., Short N.J., Alvarado Y. (2021). Quizartinib with decitabine and venetoclax (triplet) is highly active in patients with FLT3-ITD mutated acute myeloid leukemia (AML). J. Clin. Oncol..

[27] Yan H., Parsons D.W., Jin G., McLendon R., Rasheed B.A., Yuan W., Kos I., Batinic-Haberle I., Jones S., Riggins G.J. (2009). IDH1 and IDH2 mutations in gliomas. N. Engl. J. Med..

[28] Mardis E.R., Ding L., Dooling D.J., Larson D.E., McLellan M.D., Chen K., Koboldt D.C., Fulton R.S., Delehaunty K.D., McGrath S.D. (2009). Recurring mutations found by sequencing an acute myeloid leukemia genome. N. Engl. J. Med..

[29] DiNardo C.D., Ravandi F., Agresta S., Konopleva M., Takahashi K., Kadia T., Routbort M., Patel K.P., Mark B., Pierce S. (2015). Characteristics, clinical outcome, and prognostic significance of IDH mutations in AML. Am. J. Hematol..

[30] Dang L., White D.W., Gross S., Bennett B.D., Bittinger M.A., Driggers E.M., Fantin V.R., Jang H.G., Jin S., Keenan M.C. (2009). Cancer-associated IDH1 mutations produce 2-hydroxyglutarate. Nature.

[31] Ward P.S., Patel J., Wise D.R., Abdel-Wahab O., Bennett B.D., Coller H.A., Cross J.R., Fantin V.R., Hedvat C.V., Perl A.E. (2010). The common feature of leukemia-associated IDH1 and IDH2 mutations is a neomorphic enzyme activity converting alpha-ketoglutarate to 2-hydroxyglutarate. Cancer Cell.

[32] Ward P.S., Lu C., Cross J.R., Abdel-Wahab O., Levine R.L., Schwartz G.K., Thompson C.B. (2013). The potential for isocitrate dehydrogenase mutations to produce 2-hydroxyglutarate depends on allele specificity and subcellular compartmentalization. J. Biol. Chem..

[33] Figueroa M.E., Abdel-Wahab O., Lu C., Ward P.S., Patel J., Shih A., Li Y., Bhagwat N., Vasanthakumar A., Fernandez H.F. (2010). Leukemic IDH1 and IDH2 mutations result in a hypermethylation phenotype, disrupt TET2 function, and impair hematopoietic differentiation. Cancer Cell.

[34] Dunlap J.B., Leonard J., Rosenberg M., Cook R., Press R., Fan G., Raess P.W., Druker B.J., Traer E. (2019). The combination of NPM1, DNMT3A, and IDH1/2 mutations leads to inferior overall survival in AML. Am. J. Hematol..

[35] Papaemmanuil E., Gerstung M., Bullinger L., Gaidzik V.I., Paschka P., Roberts N.D., Potter N.E., Heuser M., Thol F., Bolli N. (2016). Genomic Classification and Prognosis in Acute Myeloid Leukemia. N. Engl. J. Med..

[36] Stein E.M., DiNardo C.D., Fathi A.T., Pollyea D.A., Stone R.M., Altman J.K., Roboz G.J., Patel M.R., Collins R., Flinn I.W. (2019). Molecular remission and response patterns in patients with mutant-IDH2 acute myeloid leukemia treated with enasidenib. Blood.

[37] DiNardo C.D., Stein E.M., de Botton S., Roboz G.J., Altman J.K., Mims A.S., Swords R., Collins R.H., Mannis G.N., Pollyea D.A. (2018). Durable Remissions with Ivosidenib in IDH1-Mutated Relapsed or Refractory AML. N. Engl. J. Med..

[38] Roboz G.J., DiNardo C.D., Stein E.M., de Botton S., Mims A.S., Prince G.T., Altman J.K., Arellano M.L., Donnellan W., Erba H.P. (2020). Ivosidenib induces deep durable remissions in patients with newly diagnosed IDH1-mutant acute myeloid leukemia. Blood.

[39] Yen K., Travins J., Wang F., David M.D., Artin E., Straley K., Padyana A., Gross S., DeLaBarre B., Tobin E. (2017). AG-221, a First-in-Class Therapy Targeting Acute Myeloid Leukemia Harboring Oncogenic IDH2 Mutations. Cancer Discov..

[40] Rohle D., Popovici-Muller J., Palaskas N., Turcan S., Grommes C., Campos C., Tsoi J., Clark O., Oldrini B., Komisopoulou E. (2013). An inhibitor of mutant IDH1 delays growth and promotes differentiation of glioma cells. Science.

[41] Fathi A.T., DiNardo C.D., Kline I., Kenvin L., Gupta I., Attar E.C., Stein E.M., de Botton S., Investigators A.C.S. (2018). Differentiation Syndrome Associated With Enasidenib, a Selective Inhibitor of Mutant Isocitrate Dehydrogenase 2: Analysis of a Phase 1/2 Study. JAMA Oncol..

[42] Becker J.S., Fathi A.T. (2020). Targeting IDH Mutations in AML: Wielding the Double-edged Sword of Differentiation. Curr. Cancer Drug Targets.

[43] Choe S., Wang H., DiNardo C.D., Stein E.M., de Botton S., Roboz G.J., Altman J.K., Mims A.S., Watts J.M., Pollyea D.A. (2020). Molecular mechanisms mediating relapse following ivosidenib monotherapy in IDH1-mutant relapsed or refractory AML. Blood Adv..

[44] Intlekofer A.M., Shih A.H., Wang B., Nazir A., Rustenburg A.S., Albanese S.K., Patel M., Famulare C., Correa F.M., Takemoto N. (2018). Acquired resistance to IDH inhibition through trans or cis dimer-interface mutations. Nature.

[45] Claus R., Almstedt M., Lubbert M. (2005). Epigenetic treatment of hematopoietic malignancies: In vivo targets of demethylating agents. Semin. Oncol..

[46] Dombret H., Seymour J.F., Butrym A., Wierzbowska A., Selleslag D., Jang J.H., Kumar R., Cavenagh J., Schuh A.C., Candoni A. (2015). International phase 3 study of azacitidine vs conventional care regimens in older patients with newly diagnosed AML with >30% blasts. Blood.

[47] Kantarjian H.M., Thomas X.G., Dmoszynska A., Wierzbowska A., Mazur G., Mayer J., Gau J.P., Chou W.C., Buckstein R., Cermak J. (2012). Multicenter, randomized, open-label, phase III trial of decitabine versus patient choice, with physician advice, of either supportive care or low-dose cytarabine for the treatment of older patients with newly diagnosed acute myeloid leukemia. J. Clin. Oncol..

[48] Kubasch A.S., Platzbecker U. (2018). Beyond the Edge of Hypomethylating Agents: Novel Combination Strategies for Older Adults with Advanced MDS and AML. Cancers.

[49] Wei A.H., Dohner H., Pocock C., Montesinos P., Afanasyev B., Dombret H., Ravandi F., Sayar H., Jang J.H., Porkka K. (2020). Oral Azacitidine Maintenance Therapy for Acute Myeloid Leukemia in First Remission. N. Engl. J. Med..

[50] Laille E., Shi T., Garcia-Manero G., Cogle C.R., Gore S.D., Hetzer J., Kumar K., Skikne B., MacBeth K.J. (2015). Pharmacokinetics and Pharmacodynamics with Extended Dosing of CC-486 in Patients with Hematologic Malignancies. PLoS ONE.

[51] Oliva E.N. (2020). CC-486 Reduces Hospitalization and Associated Estimated Costs in Patients with Acute Myeloid Leukemia (AML) in First Remission after Intensive Chemotherapy: Results from the QUAZAR AML-001 Maintenance Trial. Blood.

[52] de Lima M., Oran B., Champlin R.E., Papadopoulos E.B., Giralt S.A., Scott B.L., William B.M., Hetzer J., Laille E., Hubbell B. (2018). CC-486 Maintenance after Stem Cell Transplantation in Patients with Acute Myeloid Leukemia or Myelodysplastic Syndromes. Biol. Blood Marrow Transplant..

[53] Schroeder T., Czibere A., Platzbecker U., Bug G., Uharek L., Luft T., Giagounidis A., Zohren F., Bruns I., Wolschke C. (2013). Azacitidine and donor lymphocyte infusions as first salvage therapy for relapse of AML or MDS after allogeneic stem cell transplantation. Leukemia.

[54] Schroeder T., Rautenberg C. (2018). Treatment of MDS, AML and CMML Relapse after Allogeneic Blood Stem Cell Transplantation with Azacitidine, Lenalidomide and Donor Lymphocyte Infusions Results from the Second Interim Analysis of the Prospective Azalena-Trial (NCT02472691). Blood.

[55] DiNardo C.D., Pratz K.W., Letai A., Jonas B.A., Wei A.H., Thirman M., Arellano M., Frattini M.G., Kantarjian H., Popovic R. (2018). Safety and preliminary efficacy of venetoclax with decitabine or azacitidine in elderly patients with previously untreated acute myeloid leukaemia: A non-randomised, open-label, phase 1b study. Lancet Oncol..

[56] Wei A.H., Strickland S.A., Hou J.Z., Fiedler W., Lin T.L., Walter R.B., Enjeti A., Tiong I.S., Savona M., Lee S. (2019). Venetoclax Combined With Low-Dose Cytarabine for Previously Untreated Patients With Acute Myeloid Leukemia: Results From a Phase Ib/II Study. J. Clin. Oncol..

[57] Wei A.H., Montesinos P., Ivanov V., DiNardo C.D., Novak J., Laribi K., Kim I., Stevens D.A., Fiedler W., Pagoni M. (2020). Venetoclax plus LDAC for newly diagnosed AML ineligible for intensive chemotherapy: A phase 3 randomized placebo-controlled trial. Blood.

[58] DiNardo C.D., Tiong I.S., Quaglieri A., MacRaild S., Loghavi S., Brown F.C., Thijssen R., Pomilio G., Ivey A., Salmon J.M. (2020). Molecular patterns of response and treatment failure after frontline venetoclax combinations in older patients with AML. Blood.

[59] Konopleva M., Pollyea D.A., Potluri J., Chyla B., Hogdal L., Busman T., McKeegan E., Salem A.H., Zhu M., Ricker J.L. (2016). Efficacy and Biological Correlates of Response in a Phase II Study of Venetoclax Monotherapy in Patients with Acute Myelogenous Leukemia. Cancer Discov..

[60] Aldoss I., Yang D., Aribi A., Ali H., Sandhu K., Al Malki M.M., Mei M., Salhotra A., Khaled S., Nakamura R. (2018). Efficacy of the combination of venetoclax and hypomethylating agents in relapsed/refractory acute myeloid leukemia. Haematologica.

[61] Morsia E., McCullough K., Joshi M., Cook J., Alkhateeb H.B., Al-Kali A., Begna K., Elliott M., Hogan W., Litzow M. (2020). Venetoclax and hypomethylating agents in acute myeloid leukemia: Mayo Clinic series on 86 patients. Am. J. Hematol..

[62] Tenold M.E., Moskoff B.N., Benjamin D.J., Hoeg R.T., Rosenberg A.S., Abedi M., Tuscano J.M., Jonas B.A. (2021). Outcomes of Adults With Relapsed/Refractory Acute Myeloid Leukemia Treated With Venetoclax Plus Hypomethylating Agents at a Comprehensive Cancer Center. Front Oncol..

[63] Piccini M., Pilerci S., Merlini M., Grieco P., Scappini B., Bencini S., Peruzzi B., Caporale R., Signori L., Pancani F. (2021). Venetoclax-Based Regimens for Relapsed/Refractory Acute Myeloid Leukemia in a Real-Life Setting: A Retrospective Single-Center Experience. J. Clin. Med..

[64] DiNardo C.D., Rausch C.R., Benton C., Kadia T., Jain N., Pemmaraju N., Daver N., Covert W., Marx K.R., Mace M. (2018). Clinical experience with the BCL2-inhibitor venetoclax in combination therapy for relapsed and refractory acute myeloid leukemia and related myeloid malignancies. Am. J. Hematol..

[65] Schuler E., Wagner-Drouet E.M., Ajib S., Bug G., Crysandt M., Dressler S., Hausmann A., Heidenreich D., Hirschbuhl K., Hoepting M. (2021). Treatment of myeloid malignancies relapsing after allogeneic hematopoietic stem cell transplantation with venetoclax and hypomethylating agents-a retrospective multicenter analysis on behalf of the German Cooperative Transplant Study Group. Ann. Hematol..

[66] Byrne M., Danielson N., Sengsayadeth S., Rasche A., Culos K., Gatwood K., Wyatt H., Chinratanalab W., Dholaria B., Ferrell P.B. (2020). The use of venetoclax-based salvage therapy for post-hematopoietic cell transplantation relapse of acute myeloid leukemia. Am. J. Hematol..

[67] Sandhu K.S., Dadwal S., Yang D., Mei M., Palmer J., Salhotra A., Al Malki M., Aribi A., Ali H., Khaled S. (2020). Outcome of Allogeneic Hematopoietic Cell Transplantation after Venetoclax and Hypomethylating Agent Therapy for Acute Myelogenous Leukemia. Biol. Blood Marrow Transplant..

[68] DiNardo C.D., Lachowiez C.A., Takahashi K., Loghavi S., Xiao L., Kadia T., Daver N., Adeoti M., Short N.J., Sasaki K. (2021). Venetoclax Combined With FLAG-IDA Induction and Consolidation in Newly Diagnosed and Relapsed or Refractory Acute Myeloid Leukemia. J. Clin. Oncol..

[69] Ma J., Zhao S., Qiao X., Knight T., Edwards H., Polin L., Kushner J., Dzinic S.H., White K., Wang G. (2019). Inhibition of Bcl-2 Synergistically Enhances the Antileukemic Activity of Midostaurin and Gilteritinib in Preclinical Models of FLT3-Mutated Acute Myeloid Leukemia. Clin. Cancer Res..

[70] Rahmani M., Aust M.M., Attkisson E., Williams D.C., Ferreira-Gonzalez A., Grant S. (2012). Inhibition of Bcl-2 antiapoptotic members by obatoclax potently enhances sorafenib-induced apoptosis in human myeloid leukemia cells through a Bim-dependent process. Blood.

[71] Ravandi F., Alattar M.L., Grunwald M.R., Rudek M.A., Rajkhowa T., Richie M.A., Pierce S., Daver N., Garcia-Manero G., Faderl S. (2013). Phase 2 study of azacytidine plus sorafenib in patients with acute myeloid leukemia and FLT-3 internal tandem duplication mutation. Blood.

[72] Perl A.D.N., Pratz K., Dilley K. (2019). Venetoclax in Combination with Gilteritinib in Patients with Relapsed/Refractory Acute Myeloid Leukemia: A Phase 1b Study. Blood.

[73] Qin T., Jelinek J., Si J., Shu J., Issa J.P. (2009). Mechanisms of resistance to 5-aza-2′-deoxycytidine in human cancer cell lines. Blood.

[74] Hollenbach P.W., Nguyen A.N., Brady H., Williams M., Ning Y., Richard N., Krushel L., Aukerman S.L., Heise C., MacBeth K.J. (2010). A comparison of azacitidine and decitabine activities in acute myeloid leukemia cell lines. PLoS ONE.

[75] Leone G., D’Alo F., Zardo G., Voso M.T., Nervi C. (2008). Epigenetic treatment of myelodysplastic syndromes and acute myeloid leukemias. Curr. Med. Chem..

[76] Saliba A.N., John A.J., Kaufmann S.H. (2021). Resistance to venetoclax and hypomethylating agents in acute myeloid leukemia. Cancer Drug Resist..

[77] Hummel-Eisenbeiss J., Hascher A., Hals P.A., Sandvold M.L., Muller-Tidow C., Lyko F., Rius M. (2013). The role of human equilibrative nucleoside transporter 1 on the cellular transport of the DNA methyltransferase inhibitors 5-azacytidine and CP-4200 in human leukemia cells. Mol. Pharmacol..

[78] Wu P., Geng S., Weng J., Deng C., Lu Z., Luo C., Du X. (2015). The hENT1 and DCK genes underlie the decitabine response in patients with myelodysplastic syndrome. Leuk Res..

[79] Stresemann C., Lyko F. (2008). Modes of action of the DNA methyltransferase inhibitors azacytidine and decitabine. Int. J. Cancer.

[80] Zauri M., Berridge G., Thezenas M.L., Pugh K.M., Goldin R., Kessler B.M., Kriaucionis S. (2015). CDA directs metabolism of epigenetic nucleosides revealing a therapeutic window in cancer. Nature.

[81] Coombs C.C., Sallman D.A., Devlin S.M., Dixit S., Mohanty A., Knapp K., Al Ali N.H., Lancet J.E., List A.F., Komrokji R.S. (2016). Mutational correlates of response to hypomethylating agent therapy in acute myeloid leukemia. Haematologica.

[82] Cedena M.T., Rapado I., Santos-Lozano A., Ayala R., Onecha E., Abaigar M., Such E., Ramos F., Cervera J., Diez-Campelo M. (2017). Mutations in the DNA methylation pathway and number of driver mutations predict response to azacitidine in myelodysplastic syndromes. Oncotarget.

[83] Traina F., Visconte V., Elson P., Tabarroki A., Jankowska A.M., Hasrouni E., Sugimoto Y., Szpurka H., Makishima H., O’Keefe C.L. (2014). Impact of molecular mutations on treatment response to DNMT inhibitors in myelodysplasia and related neoplasms. Leukemia.

[84] Itzykson R., Kosmider O., Cluzeau T., Mansat-De Mas V., Dreyfus F., Beyne-Rauzy O., Quesnel B., Vey N., Gelsi-Boyer V., Raynaud S. (2011). Impact of TET2 mutations on response rate to azacitidine in myelodysplastic syndromes and low blast count acute myeloid leukemias. Leukemia.

[85] Craddock C.F., Houlton A.E., Quek L.S., Ferguson P., Gbandi E., Roberts C., Metzner M., Garcia-Martin N., Kennedy A., Hamblin A. (2017). Outcome of Azacitidine Therapy in Acute Myeloid Leukemia Is not Improved by Concurrent Vorinostat Therapy but Is Predicted by a Diagnostic Molecular Signature. Clin. Cancer Res..

[86] Stasik S., Eckardt J.N., Kramer M., Rollig C., Kramer A., Scholl S., Hochhaus A., Crysandt M., Brummendorf T.H., Naumann R. (2021). Impact of PTPN11 mutations on clinical outcome analyzed in 1529 patients with acute myeloid leukemia. Blood Adv..

[87] Middeke J.M., Teipel R., Rollig C., Stasik S., Zebisch A., Sill H., Kramer M., Scholl S., Hochhaus A., Jost E. (2021). Decitabine treatment in 311 patients with acute myeloid leukemia: Outcome and impact of TP53 mutations—A registry based analysis. Leuk Lymphoma.

[88] Certo M., Del Gaizo Moore V., Nishino M., Wei G., Korsmeyer S., Armstrong S.A., Letai A. (2006). Mitochondria primed by death signals determine cellular addiction to antiapoptotic BCL-2 family members. Cancer Cell.

[89] Adams J.M., Cory S. (2018). The BCL-2 arbiters of apoptosis and their growing role as cancer targets. Cell Death Differ..

[90] Czabotar P.E., Lessene G., Strasser A., Adams J.M. (2014). Control of apoptosis by the BCL-2 protein family: Implications for physiology and therapy. Nat. Rev. Mol. Cell Biol..

[91] Leverson J.D., Sampath D., Souers A.J., Rosenberg S.H., Fairbrother W.J., Amiot M., Konopleva M., Letai A. (2017). Found in Translation: How Preclinical Research Is Guiding the Clinical Development of the BCL2-Selective Inhibitor Venetoclax. Cancer Discov..

[92] Kaufmann S.H., Karp J.E., Svingen P.A., Krajewski S., Burke P.J., Gore S.D., Reed J.C. (1998). Elevated expression of the apoptotic regulator Mcl-1 at the time of leukemic relapse. Blood.

[93] Pan R., Ruvolo V., Mu H., Leverson J.D., Nichols G., Reed J.C., Konopleva M., Andreeff M. (2017). Synthetic Lethality of Combined Bcl-2 Inhibition and p53 Activation in AML: Mechanisms and Superior Antileukemic Efficacy. Cancer Cell.

[94] Zhang H., Nakauchi Y., Kohnke T., Stafford M., Bottomly D., Thomas R., Wilmot B., McWeeney S.K., Majeti R., Tyner J.W. (2020). Integrated analysis of patient samples identifies biomarkers for venetoclax efficacy and combination strategies in acute myeloid leukemia. Nat. Cancer.

[95] Han L., Zhang Q., Dail M., Shi C., Cavazos A., Ruvolo V.R., Zhao Y., Kim E., Rahmani M., Mak D.H. (2020). Concomitant targeting of BCL2 with venetoclax and MAPK signaling with cobimetinib in acute myeloid leukemia models. Haematologica.

[96] Nechiporuk T., Kurtz S.E., Nikolova O., Liu T., Jones C.L., D’Alessandro A., Culp-Hill R., d’Almeida A., Joshi S.K., Rosenberg M. (2019). The TP53 Apoptotic Network Is a Primary Mediator of Resistance to BCL2 Inhibition in AML Cells. Cancer Discov..

[97] Neel B.G., Gu H., Pao L. (2003). The ‘Shp’ing news: SH2 domain-containing tyrosine phosphatases in cell signaling. Trends Biochem. Sci..

[98] Mohi M.G., Neel B.G. (2007). The role of Shp2 (PTPN11) in cancer. Curr. Opin. Genet. Dev..

[99] Tartaglia M., Niemeyer C.M., Fragale A., Song X., Buechner J., Jung A., Hahlen K., Hasle H., Licht J.D., Gelb B.D. (2003). Somatic mutations in PTPN11 in juvenile myelomonocytic leukemia, myelodysplastic syndromes and acute myeloid leukemia. Nat. Genet..

[100] Nabinger S.C., Li X.J., Ramdas B., He Y., Zhang X., Zeng L., Richine B., Bowling J.D., Fukuda S., Goenka S. (2013). The protein tyrosine phosphatase, Shp2, positively contributes to FLT3-ITD-induced hematopoietic progenitor hyperproliferation and malignant disease in vivo. Leukemia.

[101] Chen L., Chen W., Mysliwski M., Serio J., Ropa J., Abulwerdi F.A., Chan R.J., Patel J.P., Tallman M.S., Paietta E. (2015). Mutated Ptpn11 alters leukemic stem cell frequency and reduces the sensitivity of acute myeloid leukemia cells to Mcl1 inhibition. Leukemia.

[102] Yang Z., Li Y., Yin F., Chan R.J. (2008). Activating PTPN11 mutants promote hematopoietic progenitor cell-cycle progression and survival. Exp. Hematol..

[103] Zhu X.Z., Yu Y.Z., Fang Y.M., Liang Y., Lu Q.H., Xu R.Z. (2005). Overexpression of Shp-2 is associated with the unlimited growth and apoptosis resistance of p210 bcr-abl-mediated chronic myeloid leukemia. Zhonghua Yi Xue Za Zhi.

[104] Chyla B., Daver N., Doyle K., McKeegan E., Huang X., Ruvolo V., Wang Z., Chen K., Souers A., Leverson J. (2018). Genetic Biomarkers Of Sensitivity and Resistance to Venetoclax Monotherapy in Patients With Relapsed Acute Myeloid Leukemia. Am. J. Hematol..

[105] Li L., Modi H., McDonald T., Rossi J., Yee J.K., Bhatia R. (2011). A critical role for SHP2 in STAT5 activation and growth factor-mediated proliferation, survival, and differentiation of human CD34+ cells. Blood.

[106] Singh Mali R.L.E.A. (2017). FLT3-ITD Activation Mediates Resistance to the BCL-2 Selective Antagonist, Venetoclax, in FLT3-ITD Mutant AML Models. Blood.

[107] Chen X., Glytsou C., Zhou H., Narang S., Reyna D.E., Lopez A., Sakellaropoulos T., Gong Y., Kloetgen A., Yap Y.S. (2019). Targeting Mitochondrial Structure Sensitizes Acute Myeloid Leukemia to Venetoclax Treatment. Cancer Discov..

[108] Sharon D., Cathelin S., Mirali S., Di Trani J.M., Yanofsky D.J., Keon K.A., Rubinstein J.L., Schimmer A.D., Ketela T., Chan S.M. (2019). Inhibition of mitochondrial translation overcomes venetoclax resistance in AML through activation of the integrated stress response. Sci. Transl. Med..

[109] Pollyea D.A., Stevens B.M., Jones C.L., Winters A., Pei S., Minhajuddin M., D’Alessandro A., Culp-Hill R., Riemondy K.A., Gillen A.E. (2018). Venetoclax with azacitidine disrupts energy metabolism and targets leukemia stem cells in patients with acute myeloid leukemia. Nat. Med..

[110] Akgul C. (2009). Mcl-1 is a potential therapeutic target in multiple types of cancer. Cell Mol. Life. Sci..

[111] Luedtke D.A., Niu X., Pan Y., Zhao J., Liu S., Edwards H., Chen K., Lin H., Taub J.W., Ge Y. (2017). Inhibition of Mcl-1 enhances cell death induced by the Bcl-2-selective inhibitor ABT-199 in acute myeloid leukemia cells. Signal Transduct. Target Ther..

[112] Kotschy A., Szlavik Z., Murray J., Davidson J., Maragno A.L., Le Toumelin-Braizat G., Chanrion M., Kelly G.L., Gong J.N., Moujalled D.M. (2016). The MCL1 inhibitor S63845 is tolerable and effective in diverse cancer models. Nature.

[113] Li Z., He S., Look A.T. (2019). The MCL1-specific inhibitor S63845 acts synergistically with venetoclax/ABT-199 to induce apoptosis in T-cell acute lymphoblastic leukemia cells. Leukemia.

[114] Ramsey H.E., Fischer M.A., Lee T., Gorska A.E., Arrate M.P., Fuller L., Boyd K.L., Strickland S.A., Sensintaffar J., Hogdal L.J. (2018). A Novel MCL1 Inhibitor Combined with Venetoclax Rescues Venetoclax-Resistant Acute Myelogenous Leukemia. Cancer Discov..

[115] Roberts A.W., Wei A.H., Huang D.C. (2021). BCL2 and MCL1 inhibitors for hematologic malignancies. Blood.

[116] Kasper S., Breitenbuecher F., Heidel F., Hoffarth S., Markova B., Schuler M., Fischer T. (2012). Targeting MCL-1 sensitizes FLT3-ITD-positive leukemias to cytotoxic therapies. Blood Cancer J..

[117] Chen J., Jin S., Abraham V., Huang X., Liu B., Mitten M.J., Nimmer P., Lin X., Smith M., Shen Y. (2011). The Bcl-2/Bcl-X(L)/Bcl-w inhibitor, navitoclax, enhances the activity of chemotherapeutic agents in vitro and in vivo. Mol. Cancer Ther..

[118] Tron A.E., Belmonte M.A., Adam A., Aquila B.M., Boise L.H., Chiarparin E., Cidado J., Embrey K.J., Gangl E., Gibbons F.D. (2018). Discovery of Mcl-1-specific inhibitor AZD5991 and preclinical activity in multiple myeloma and acute myeloid leukemia. Nat. Commun..

[119] Knight T., Edwards H., Taub J.W., Ge Y. (2019). Evaluating venetoclax and its potential in treatment-naive acute myeloid leukemia. Cancer Manag. Res..

[120] Mandal R., Becker S., Strebhardt K. (2021). Targeting CDK9 for Anti-Cancer Therapeutics. Cancers.

[121] Bogenberger J., Whatcott C., Hansen N., Delman D., Shi C.X., Kim W., Haws H., Soh K., Lee Y.S., Peterson P. (2017). Combined venetoclax and alvocidib in acute myeloid leukemia. Oncotarget.

[122] Daver N.G., Pollyea D.A., Garcia J.S., Jonas B.A., Yee K.W., Fenaux P., Assouline S., Vey N., Olin R., Roboz G.J. (2018). Safety, Efficacy, Pharmacokinetic (PK) and Biomarker Analyses of BCL2 Inhibitor Venetoclax (Ven) Plus MDM2 Inhibitor Idasanutlin (idasa) in Patients (pts) with Relapsed or Refractory (R/R) AML: A Phase Ib, Non-Randomized, Open-Label Study. Blood.

[123] Lainez-González D., Serrano-López J., Alonso-Domínguez J.M. (2021). Understanding the Hedgehog Signaling Pathway in Acute Myeloid Leukemia Stem Cells: A Necessary Step toward a Cure. Biology.

[124] Huang K., Sun Z., Ding B., Jiang X., Wang Z., Zhu Y., Meng F. (2019). Suppressing Hedgehog signaling reverses drug resistance of refractory acute myeloid leukemia. Onco Targets Ther..

[125] Tibes R., Al-Kali A., Oliver G.R., Delman D.H., Hansen N., Bhagavatula K., Mohan J., Rakhshan F., Wood T., Foran J.M. (2015). The Hedgehog pathway as targetable vulnerability with 5-azacytidine in myelodysplastic syndrome and acute myeloid leukemia. J Hematol. Oncol..

[126] Cortes J.E., Heidel F.H., Hellmann A., Fiedler W., Smith B.D., Robak T., Montesinos P., Pollyea D.A., DesJardins P., Ottmann O. (2019). Randomized comparison of low dose cytarabine with or without glasdegib in patients with newly diagnosed acute myeloid leukemia or high-risk myelodysplastic syndrome. Leukemia.

[127] Issa G.C., Ravandi F., DiNardo C.D., Jabbour E., Kantarjian H.M., Andreeff M. (2021). Therapeutic implications of menin inhibition in acute leukemias. Leukemia.

[128] Schoch C., Schnittger S., Klaus M., Kern W., Hiddemann W., Haferlach T. (2003). AML with 11q23/MLL abnormalities as defined by the WHO classification: Incidence, partner chromosomes, FAB subtype, age distribution, and prognostic impact in an unselected series of 1897 cytogenetically analyzed AML cases. Blood.

[129] Sun Q.Y., Ding L.W., Tan K.T., Chien W., Mayakonda A., Lin D.C., Loh X.Y., Xiao J.F., Meggendorfer M., Alpermann T. (2017). Ordering of mutations in acute myeloid leukemia with partial tandem duplication of MLL (MLL-PTD). Leukemia.

[130] Matkar S.S., Thiel A.T., Hua X. (2013). Menin: A scaffold protein that controls gene expression and cell signaling. Trends Biochem. Sci..

[131] Wang E.S., Altman J.K., Pettit K., De Botton S., Walter R.P., Fenaux P., Burrows F., Tomkinson B.E., Martell B., Fathi A.T. (2020). Preliminary Data on a Phase 1/2A First in Human Study of the Menin-KMT2A (MLL) Inhibitor KO-539 in Patients with Relapsed or Refractory Acute Myeloid Leukemia. Blood.

[132] McGeehan J. A first-in-class Menin-MLL1 antagonist for the treatment of MLL-r and NPM1 mutant leukemias. Proceedings of the AACR Annual Meeting.

[133] Morgado E., Albouhair S.p., Lavau C. (2007). Flt3 is dispensable to the Hoxa9/Meis1 leukemogenic cooperation. Blood.

[134] Dzama M.M., Steiner M., Rausch J., Sasca D., Schönfeld J., Kunz K., Taubert M.C., McGeehan G.M., Chen C.W., Mupo A. (2020). Synergistic Targeting of FLT3 Mutations in AML via Combined Menin-MLL and FLT3 Inhibition. Blood.

[135] Haferlach C., Dicker F., Herholz H., Schnittger S., Kern W., Haferlach T. (2008). Mutations of the TP53 gene in acute myeloid leukemia are strongly associated with a complex aberrant karyotype. Leukemia.

[136] Konopleva M., Martinelli G., Daver N., Papayannidis C., Wei A., Higgins B., Ott M., Mascarenhas J., Andreeff M. (2020). MDM2 inhibition: An important step forward in cancer therapy. Leukemia.

[137] Andreeff M., Kelly K.R., Yee K., Assouline S., Strair R., Popplewell L., Bowen D., Martinelli G., Drummond M.W., Vyas P. (2016). Results of the Phase I Trial of RG7112, a Small-Molecule MDM2 Antagonist in Leukemia. Clin. Cancer Res..

[138] Montesinos P., Beckermann B.M., Catalani O., Esteve J., Gamel K., Konopleva M.Y., Martinelli G., Monnet A., Papayannidis C., Park A. (2020). MIRROS: A randomized, placebo-controlled, Phase III trial of cytarabine ± idasanutlin in relapsed or refractory acute myeloid leukemia. Future Oncol..

[139] Daver N.G., Garcia J.S., Jonas B.A., Kelly K.R., Assouline S., Brandwein J.M., Fenaux P., Olin R.L., Martinelli G., Paolini S. (2019). Updated Results from the Venetoclax (Ven) in Combination with Idasanutlin (Idasa) Arm of a Phase 1b Trial in Elderly Patients (Pts) with Relapsed or Refractory (R/R) AML Ineligible for Cytotoxic Chemotherapy. Blood.

[140] Sallman D.A., Borate U., Cull E.H., Donnellan W.B., Komrokji R.S., Steidl U.G., Corvez M.M., Payton M., Annis D.A., Pinchasik D. (2018). Phase 1/1b Study of the Stapled Peptide ALRN-6924, a Dual Inhibitor of MDMX and MDM2, As Monotherapy or in Combination with Cytarabine for the Treatment of Relapsed/Refractory AML and Advanced MDS with TP53 Wild-Type. Blood.

[141] Erba H.P., Becker P.S., Shami P.J., Grunwald M.R., Flesher D.L., Zhu M., Rasmussen E., Henary H.A., Anderson A.A., Wang E.S. (2019). Phase 1b study of the MDM2 inhibitor AMG 232 with or without trametinib in relapsed/refractory acute myeloid leukemia. Blood Adv..

[142] Zeidan A.M., Ridinger M., Lin T.L., Becker P.S., Schiller G.J., Patel P.A., Spira A.I., Tsai M.L., Samuelsz E., Silberman S.L. (2020). A Phase Ib Study of Onvansertib, a Novel Oral PLK1 Inhibitor, in Combination Therapy for Patients with Relapsed or Refractory Acute Myeloid Leukemia. Clin. Cancer Res..

[143] Sallman D.A., DeZern A.E., Garcia-Manero G., Steensma D.P., Roboz G.J., Sekeres M.A., Cluzeau T., Sweet K.L., McLemore A., McGraw K.L. (2021). Eprenetapopt (APR-246) and Azacitidine in TP53-Mutant Myelodysplastic Syndromes. J. Clin. Oncol..

[144] Menghrajani K., Cai S.F., Devlin S.M., Armstrong S.A., Piekartz R., Rudek M., Stein E.M. (2019). A Phase Ib/II Study of the Histone Methyltransferase Inhibitor Pinometostat in Combination with Azacitidine in Patients with 11q23-Rearranged Acute Myeloid Leukemia. Blood.

[145] Borthakur G., Odenike O., Aldoss I., Rizzieri D.A., Prebet T., Chen C., Popovic R., Modi D.A., Joshi R.H., Wolff J.E. (2021). A phase 1 study of the pan-bromodomain and extraterminal inhibitor mivebresib (ABBV-075) alone or in combination with venetoclax in patients with relapsed/refractory acute myeloid leukemia. Cancer.

[146] Bhatnagar B., Zhao Q., Mims A.S., Vasu S., Behbehani G.K., Larkin K., Blachly J.S., Blum W., Klisovic R.B., Ruppert A.S. (2020). Selinexor in combination with decitabine in patients with acute myeloid leukemia: Results from a phase 1 study. Leuk Lymphoma.

[147] Zeidner J.F., Lee D.J., Frattini M., Fine G.D., Costas J., Kolibaba K., Anthony S.P., Bearss D., Smith B.D. (2021). Phase I Study of Alvocidib Followed by 7+3 (Cytarabine + Daunorubicin) in Newly Diagnosed Acute Myeloid Leukemia. Clin. Cancer Res..

[148] Castaigne S., Pautas C., Terré C., Raffoux E., Bordessoule D., Bastie J.N., Legrand O., Thomas X., Turlure P., Reman O. (2012). Effect of gemtuzumab ozogamicin on survival of adult patients with de-novo acute myeloid leukaemia (ALFA-0701): A randomised, open-label, phase 3 study. Lancet.

[149] Lambert J., Pautas C., Terré C., Raffoux E., Turlure P., Caillot D., Legrand O., Thomas X., Gardin C., Gogat-Marchant K. (2019). Gemtuzumab ozogamicin for de novo acute myeloid leukemia: Final efficacy and safety updates from the open-label, phase III ALFA-0701 trial. Haematologica.

[150] Lamba J.K., Chauhan L., Shin M., Loken M.R., Pollard J.A., Wang Y.C., Ries R.E., Aplenc R., Hirsch B.A., Raimondi S.C. (2017). CD33 Splicing Polymorphism Determines Gemtuzumab Ozogamicin Response in De Novo Acute Myeloid Leukemia: Report From Randomized Phase III Children’s Oncology Group Trial AAML0531. J. Clin. Oncol..

[151] Gale R.E., Popa T., Wright M., Khan N., Freeman S.D., Burnett A.K., Russell N.H., Hills R.K., Linch D.C. (2018). No evidence that CD33 splicing SNP impacts the response to GO in younger adults with AML treated on UK MRC/NCRI trials. Blood.

[152] Petersdorf S.H., Kopecky K.J., Slovak M., Willman C., Nevill T., Brandwein J., Larson R.A., Erba H.P., Stiff P.J., Stuart R.K. (2013). A phase 3 study of gemtuzumab ozogamicin during induction and postconsolidation therapy in younger patients with acute myeloid leukemia. Blood.

[153] Amadori S., Suciu S., Stasi R., Salih H.R., Selleslag D., Muus P., Fabritiis P.D., Venditti A., Ho A.D., Lübbert M. (2013). Sequential Combination of Gemtuzumab Ozogamicin and Standard Chemotherapy in Older Patients With Newly Diagnosed Acute Myeloid Leukemia: Results of a Randomized Phase III Trial by the EORTC and GIMEMA Consortium (AML-17). J. Clin. Oncol..

[154] Schlenk R.F., Paschka P., Krzykalla J., Weber D., Kapp-Schwoerer S., Gaidzik V.I., Leis C., Fiedler W., Kindler T., Schroeder T. (2020). Gemtuzumab Ozogamicin in NPM1-Mutated Acute Myeloid Leukemia: Early Results From the Prospective Randomized AMLSG 09-09 Phase III Study. J. Clin. Oncol..

[155] Kapp-Schwoerer S., Weber D., Corbacioglu A., Gaidzik V.I., Paschka P., Krönke J., Theis F., Rücker F.G., Teleanu M.V., Panina E. (2020). Impact of gemtuzumab ozogamicin on MRD and relapse risk in patients with NPM1-mutated AML: Results from the AMLSG 09-09 trial. Blood.

[156] Kantarjian H., Stein A., Gökbuget N., Fielding A.K., Schuh A.C., Ribera J.-M., Wei A., Dombret H., Foà R., Bassan R. (2017). Blinatumomab versus Chemotherapy for Advanced Acute Lymphoblastic Leukemia. N. Engl. J. Med..

[157] Maude S.L., Laetsch T.W., Buechner J., Rives S., Boyer M., Bittencourt H., Bader P., Verneris M.R., Stefanski H.E., Myers G.D. (2018). Tisagenlecleucel in Children and Young Adults with B-Cell Lymphoblastic Leukemia. N. Engl. J. Med..

[158] Graf C., Heidel F., Tenzer S., Radsak M.P., Solem F.K., Britten C.M., Huber C., Fischer T., Wölfel T. (2007). A neoepitope generated by an FLT3 internal tandem duplication (FLT3-ITD) is recognized by leukemia-reactive autologous CD8+ T cells. Blood.

[159] Daver N., Alotaibi A.S., Bücklein V., Subklewe M. (2021). T-cell-based immunotherapy of acute myeloid leukemia: Current concepts and future developments. Leukemia.

[160] Ravandi F., Walter R.B., Subklewe M., Buecklein V., Jongen-Lavrencic M., Paschka P., Ossenkoppele G.J., Kantarjian H.M., Hindoyan A., Agarwal S.K. (2020). Updated results from phase I dose-escalation study of AMG 330, a bispecific T-cell engager molecule, in patients with relapsed/refractory acute myeloid leukemia (R/R AML). J. Clin. Oncol..

[161] Subklewe M., Stein A., Walter R.B., Bhatia R., Wei A.H., Ritchie D., Bücklein V., Vachhani P., Dai T., Hindoyan A. (2019). Preliminary Results from a Phase 1 First-in-Human Study of AMG 673, a Novel Half-Life Extended (HLE) Anti-CD33/CD3 BiTE^®^ (Bispecific T-Cell Engager) in Patients with Relapsed/Refractory (R/R) Acute Myeloid Leukemia (AML). Blood.

[162] Uy G.L., Aldoss I., Foster M.C., Sayre P.H., Wieduwilt M.J., Advani A.S., Godwin J.E., Arellano M.L., Sweet K.L., Emadi A. (2021). Flotetuzumab as salvage immunotherapy for refractory acute myeloid leukemia. Blood.

[163] Ravandi F., Bashey A., Stock W., Foran J.M., Mawad R., Egan D., Blum W., Yang A., Pastore A., Johnson C. (2020). Complete Responses in Relapsed/Refractory Acute Myeloid Leukemia (AML) Patients on a Weekly Dosing Schedule of Vibecotamab (XmAb14045), a CD123 x CD3 T Cell-Engaging Bispecific Antibody; Initial Results of a Phase 1 Study. Blood.

[164] Brauchle B., Goldstein R.L., Karbowski C.M., Henn A., Li C.-M., Bücklein V.L., Krupka C., Boyle M.C., Koppikar P., Haubner S. (2020). Characterization of a Novel FLT3 BiTE Molecule for the Treatment of Acute Myeloid Leukemia. Mol. Cancer Ther..

[165] Schmidt-Arras D., Böhmer S.A., Koch S., Müller J.P., Blei L., Cornils H., Bauer R., Korasikha S., Thiede C., Böhmer F.D. (2009). Anchoring of FLT3 in the endoplasmic reticulum alters signaling quality. Blood.

[166] van Loo P.F., Hangalapura B.N., Thordardottir S., Gibbins J.D., Veninga H., Hendriks L.J.A., Kramer A., Roovers R.C., Leenders M., de Kruif J. (2019). MCLA-117, a CLEC12AxCD3 bispecific antibody targeting a leukaemic stem cell antigen, induces T cell-mediated AML blast lysis. Expert Opin. Biol. Ther..

[167] Wang Z., Chen J., Wang M., Zhang L., Yu L. (2021). One Stone, Two Birds: The Roles of Tim-3 in Acute Myeloid Leukemia. Front. Immunol..

[168] Gonçalves Silva I., Rüegg L., Gibbs B.F., Bardelli M., Fruehwirth A., Varani L., Berger S.M., Fasler-Kan E., Sumbayev V.V. (2016). The immune receptor Tim-3 acts as a trafficker in a Tim-3/galectin-9 autocrine loop in human myeloid leukemia cells. Oncoimmunology.

[169] Kikushige Y., Miyamoto T., Yuda J., Jabbarzadeh-Tabrizi S., Shima T., Takayanagi S., Niiro H., Yurino A., Miyawaki K., Takenaka K. (2015). A TIM-3/Gal-9 Autocrine Stimulatory Loop Drives Self-Renewal of Human Myeloid Leukemia Stem Cells and Leukemic Progression. Cell Stem. Cell.

[170] Guzman M.L., Neering S.J., Upchurch D., Grimes B., Howard D.S., Rizzieri D.A., Luger S.M., Jordan C.T. (2001). Nuclear factor-kappaB is constitutively activated in primitive human acute myelogenous leukemia cells. Blood.

[171] Brunner A.M., Esteve J., Porkka K., Knapper S., Vey N., Scholl S., Garcia-Manero G., Wermke M., Janssen J., Traer E. (2020). Efficacy and Safety of Sabatolimab (MBG453) in Combination with Hypomethylating Agents (HMAs) in Patients with Acute Myeloid Leukemia (AML) and High-Risk Myelodysplastic Syndrome (HR-MDS): Updated Results from a Phase 1b Study. Blood.

[172] Reck M., Rodríguez-Abreu D., Robinson A.G., Hui R., Csőszi T., Fülöp A., Gottfried M., Peled N., Tafreshi A., Cuffe S. (2016). Pembrolizumab versus Chemotherapy for PD-L1–Positive Non–Small-Cell Lung Cancer. N. Engl. J. Med..

[173] Hodi F.S., O’Day S.J., McDermott D.F., Weber R.W., Sosman J.A., Haanen J.B., Gonzalez R., Robert C., Schadendorf D., Hassel J.C. (2010). Improved Survival with Ipilimumab in Patients with Metastatic Melanoma. N. Engl. J. Med..

[174] Vereecque R., Saudemont A., Quesnel B. (2004). Cytosine arabinoside induces costimulatory molecule expression in acute myeloid leukemia cells. Leukemia.

[175] Ørskov A.D., Treppendahl M.B., Skovbo A., Holm M.S., Friis L.S., Hokland M., Grønbæk K. (2015). Hypomethylation and up-regulation of PD-1 in T cells by azacytidine in MDS/AML patients: A rationale for combined targeting of PD-1 and DNA methylation. Oncotarget.

[176] Schuster S.J., Bishop M.R., Tam C.S., Waller E.K., Borchmann P., McGuirk J.P., Jäger U., Jaglowski S., Andreadis C., Westin J.R. (2018). Tisagenlecleucel in Adult Relapsed or Refractory Diffuse Large B-Cell Lymphoma. N. Engl. J. Med..

[177] Perna F., Berman S.H., Soni R.K., Mansilla-Soto J., Eyquem J., Hamieh M., Hendrickson R.C., Brennan C.W., Sadelain M. (2017). Integrating Proteomics and Transcriptomics for Systematic Combinatorial Chimeric Antigen Receptor Therapy of AML. Cancer Cell.

[178] Liu F., Cao Y., Pinz K., Ma Y., Wada M., Chen K., Ma G., Shen J., Tse C.O., Su Y. (2018). First-in-Human CLL1-CD33 Compound CAR T Cell Therapy Induces Complete Remission in Patients with Refractory Acute Myeloid Leukemia: Update on Phase 1 Clinical Trial. Blood.

[179] He X., Feng Z., Ma J., Ling S., Cao Y., Gurung B., Wu Y., Katona B.W., O’Dwyer K.P., Siegel D.L. (2020). Bispecific and split CAR T cells targeting CD13 and TIM3 eradicate acute myeloid leukemia. Blood.

[180] Jaiswal S., Jamieson C.H., Pang W.W., Park C.Y., Chao M.P., Majeti R., Traver D., van Rooijen N., Weissman I.L. (2009). CD47 is upregulated on circulating hematopoietic stem cells and leukemia cells to avoid phagocytosis. Cell.

[181] Majeti R., Chao M.P., Alizadeh A.A., Pang W.W., Jaiswal S., Gibbs K.D., van Rooijen N., Weissman I.L. (2009). CD47 is an adverse prognostic factor and therapeutic antibody target on human acute myeloid leukemia stem cells. Cell.

[182] Sallman D.A., Malki M.A., Asch A.S., Lee D.J., Kambhampati S., Donnellan W.B., Bradley T.J., Vyas P., Jeyakumar D., Marcucci G. (2020). Tolerability and efficacy of the first-in-class anti-CD47 antibody magrolimab combined with azacitidine in MDS and AML patients: Phase Ib results. J. Clin. Oncol..

[183] Garcia-Manero G., Daver N.G., Xu J., Chao M., Chung T., Tan A., Wang V., Wei A., Vyas P., Sallman D.A. (2021). Magrolimab + azacitidine versus azacitidine + placebo in untreated higher risk (HR) myelodysplastic syndrome (MDS): The phase 3, randomized, ENHANCE study. J. Clin. Oncol..

[184] Riether C., Schürch C.M., Bührer E.D., Hinterbrandner M., Huguenin A.-L., Hoepner S., Zlobec I., Pabst T., Radpour R., Ochsenbein A.F. (2016). CD70/CD27 signaling promotes blast stemness and is a viable therapeutic target in acute myeloid leukemia. J. Exp. Med..

[185] Riether C., Pabst T., Höpner S., Bacher U., Hinterbrandner M., Banz Y., Müller R., Manz M.G., Gharib W.H., Francisco D. (2020). Targeting CD70 with cusatuzumab eliminates acute myeloid leukemia stem cells in patients treated with hypomethylating agents. Nature Med..

[186] Ochsenbein A.F., Pabst T., Höpner S., Bacher V.U., Hinterbrandner M., Banz Y., Müller R., Manz M.G., Gharib W., Francisco D. (2019). Targeting CD70 with Cusatuzumab Eliminates Acute Myeloid Leukemia Stem Cells in Humans. Blood.

[187] Sauer T., Parikh K., Sharma S., Omer B., Sedloev D., Chen Q., Angenendt L., Schliemann C., Schmitt M., Müller-Tidow C. (2021). CD70-specific CAR T cells have potent activity against acute myeloid leukemia without HSC toxicity. Blood.

[188] van Gils N., Denkers F., Smit L. (2021). Escape From Treatment; the Different Faces of Leukemic Stem Cells and Therapy Resistance in Acute Myeloid Leukemia. Front Oncol..

[189] Goardon N., Marchi E., Atzberger A., Quek L., Schuh A., Soneji S., Woll P., Mead A., Alford K.A., Rout R. (2011). Coexistence of LMPP-like and GMP-like Leukemia Stem Cells in Acute Myeloid Leukemia. Cancer Cell.

[190] Arnone M., Konantz M., Hanns P., Paczulla Stanger A.M., Bertels S., Godavarthy P.S., Christopeit M., Lengerke C. (2020). Acute Myeloid Leukemia Stem Cells: The Challenges of Phenotypic Heterogeneity. Cancers.

[191] Jung N., Dai B., Gentles A., Majeti R., Feinberg A. (2015). An LSC epigenetic signature is largely mutation independent and implicates the HOXA cluster in AML pathogenesis. Nature Commun..

[192] Ng S.W.K., Mitchell A., Kennedy J.A., Chen W.C., McLeod J., Ibrahimova N., Arruda A., Popescu A., Gupta V., Schimmer A.D. (2016). A 17-gene stemness score for rapid determination of risk in acute leukaemia. Nature.

[193] Uy G.L., Rettig M.P., Stone R.M., Konopleva M.Y., Andreeff M., McFarland K., Shannon W., Fletcher T.R., Reineck T., Eades W. (2017). A phase 1/2 study of chemosensitization with plerixafor plus G-CSF in relapsed or refractory acute myeloid leukemia. Blood Cancer J..

[194] Sheng Y., Yu C., Liu Y., Hu C., Ma R., Lu X., Ji P., Chen J., Mizukawa B., Huang Y. (2020). FOXM1 regulates leukemia stem cell quiescence and survival in MLL-rearranged AML. Nature Commun..

[195] Rouschop K.M., Dubois L.J., Keulers T.G., van den Beucken T., Lambin P., Bussink J., van der Kogel A.J., Koritzinsky M., Wouters B.G. (2013). PERK/eIF2α signaling protects therapy resistant hypoxic cells through induction of glutathione synthesis and protection against ROS. Proc. Natl. Acad. Sci USA.

[196] Stevens A.M., Xiang M., Heppler L.N., Tošić I., Jiang K., Munoz J.O., Gaikwad A.S., Horton T.M., Long X., Narayanan P. (2019). Atovaquone is active against AML by upregulating the integrated stress pathway and suppressing oxidative phosphorylation. Blood Adv...

[197] Yu X., Munoz-Sagredo L., Streule K., Muschong P., Bayer E., Walter R.J., Gutjahr J.C., Greil R., Concha M.L., Muller-Tidow C. (2021). CD44 loss of function sensitizes AML cells to the BCL-2 inhibitor venetoclax by decreasing CXCL12-driven survival cues. Blood.

